# *GEN1* as a risk factor for human congenital anomalies of the kidney and urinary tract

**DOI:** 10.1186/s40246-024-00606-8

**Published:** 2024-04-24

**Authors:** Xuanjin Du, Chunyan Wang, Jialu Liu, Minghui Yu, Haixin Ju, Shanshan Xue, Yaxin Li, Jiaojiao Liu, Rufeng Dai, Jing Chen, Yihui Zhai, Jia Rao, Xiang Wang, Yubo Sun, Lei Sun, Xiaohui Wu, Hong Xu, Qian Shen

**Affiliations:** 1 Department of Nephrology, Shanghai Kidney Development and Pediatric Kidney Disease Research Center, Children’s Hospital of Fudan University, 201102 Shanghai, China; 2 Department of Urology, Shanghai Kidney Development and Pediatric Kidney Disease Research Center, Children’s Hospital of Fudan University, 201102 Shanghai, China; 3 State Key Laboratory of Genetic Engineering and National Center for International Research of Development and Disease, Institute of Developmental Biology and Molecular Medicine, Fudan University, 200433 Shanghai, China; 4 National Key Laboratory of Kidney Diseases, 201102 Shanghai, China

**Keywords:** Congenital anomalies of the kidney and urinary tract (CAKUT), GEN1 Holliday junction 5' flap endonuclease, Point-mutant mouse model, Human

## Abstract

**Background:**

Congenital anomalies of the kidney and urinary tract (CAKUT) are prevalent birth defects. Although pathogenic CAKUT genes are known, they are insufficient to reveal the causes for all patients. Our previous studies indicated *GEN1* as a pathogenic gene of CAKUT in mice, and this study further investigated the correlation between *GEN1* and human CAKUT.

**Methods:**

In this study, DNA from 910 individuals with CAKUT was collected; 26 *GEN1* rare variants were identified, and two *GEN1* (missense) variants in a non-CAKUT group were found. Mainly due to the stability results of the predicted mutant on the website, in vitro, 10 variants (eight CAKUT, two non-CAKUT) were selected to verify mutant protein stability. In addition, mainly based on the division of the mutation site located in the functional region of the GEN1 protein, 8 variants (six CAKUT, two non-CAKUT) were selected to verify enzymatic hydrolysis, and the splice variant GEN1 (c.1071 + 3(IVS10) A > G) was selected to verify shear ability. Based on the results of in vitro experiments and higher frequency, three sites with the most significant functional change were selected to build mouse models.

**Results:**

Protein stability changed in six variants in the CAKUT group. Based on electrophoretic mobility shift assay of eight variants (six CAKUT, two non-CAKUT), the enzymatic hydrolysis and DNA-binding abilities of mutant proteins were impaired in the CAKUT group. The most serious functional damage was observed in the *Gen1* variant that produced a truncated protein. A mini-gene splicing assay showed that the variant GEN1 (c.1071 + 3(IVS10) A > G) in the CAKUT group significantly affected splicing function. An abnormal exon10 was detected in the mini-gene splicing assay. Point-mutant mouse strains were constructed (Gen1: c.1068 + 3 A > G, p.R400X, and p.T105R) based on the variant frequency in the CAKUT group and functional impairment in vitro study and CAKUT phenotypes were replicated in each.

**Conclusion:**

Overall, our findings indicated *GEN1* as a risk factor for human CAKUT.

**Supplementary Information:**

The online version contains supplementary material available at 10.1186/s40246-024-00606-8.

## Introduction

Congenital anomalies of the kidney and urinary tract (CAKUT) are common congenital malformations in newborns worldwide, with a prevalence of 3–9 per 1000 [1, 2]. More than 50% of CAKUT children require kidney replacement therapy [3–5]. The complex and diverse phenotypes involved in CAKUT include not only renal malformations, such as duplex kidneys, solitary kidneys, renal dysplasia, hydronephrosis, and horseshoe kidneys, but also various phenotypes related to the collecting system, such as a giant ureter and vesicoureteral reflux (VUR) [6, 7]. CAKUT phenotypes can occur under non-syndromic or syndromic conditions, and multiple CAKUT phenotypes can be found in a single individual [8]. The heterogeneous phenotype of CAKUT and its severe clinical consequences render it a popular topic in nephrological research.

CAKUT phenotypes are associated with complex etiologies, and genetic factors play vital roles in the pathogenesis of CAKUT [9]. Fifty genes that cause human isolated CAKUT have been identified [8, 10], most of which are involved in embryonic renal development. The critical phase of renal development can be divided into four stages, with different causative genes: ureteral bud (UB) induction (*BMP4* (*OMIM number: 112,262), EYA1 (OMIM number: 601,653), GATA3 (OMIM number: 131,320), PAX2 (OMIM number: 167,409), RET (OMIM number: 164,761), ROBO2 (OMIM number: 602,431), SIX1 (OMIM number: 601,205*), and *SIX2* (*OMIM number: 604,994*)) [11–17], epithelial mesenchymal transformation (*WNT4* (*OMIM number: 603,490*) and *FGF20* (*OMIM number: 605,558*)) [18, 19], branching morphogenesis (*AGT* (*OMIM number: 106,150*) and *AGTR* (*OMIM number: 106,165*)) [20, 21], and nephron patterning and elongation (*UMOD* (*OMIM number: 191,845*)) [22, 23]. However, monogenic causes account for only 18% of all causes [8], which is far from sufficient to reveal the causes in all patients with CAKUT, suggesting that there are many unidentified genetic causes of CAKUT that need to be further explored.

*GEN1* (*OMIM number: 612449*) is located at 2p24.2 (total length 348654 bp) and plays an important role in DNA damage repair in mammalian cells [24–29]. As a member of the Rad2/XPG family, which is conserved from yeast to humans [24, 30], GEN1 can be roughly divided into four domains, characterized by a downward palm [31]. GEN1 was originally discovered as the first human Holliday Junction (HJ) 5’ flap endonuclease in 2008 [24]. Its protease activity can be evaluated in terms of its ability to bind DNA and enzymatically disassemble HJ [31]. In our previous studies, 50% of *Gen1*^*PB/PB*^ mice displayed CAKUT phenotypes, with only 15% *Gen1* expression compared to wild-type (WT) mice. *Gen1*^*PB/+*^ mice demonstrated 60% *Gen1* expression, and 20% presented CAKUT phenotypes, mainly including, but not limited to, a duplex kidney and collection system, solitary kidney, hydronephrosis, giant ureter, and renal dysplasia [32]. The inheritance pattern of *GEN1* is still inconclusive. As shown in *Gen1*^*PB*^ mice, not all heterozygous and homozygous mice exhibit renal and urinary tract abnormalities. This is consistent with the etiological diversity and incomplete penetrance of CAKUT, reflecting the complexity of regulatory signaling during early renal development. All these results indicated the inheritance pattern in *GEN1* could be autosomal dominant and dose-dependent. Further exploration of the underlying mechanism of *Gen1* deficiency in mice showed *Gen1* mutant mice exhibit CAKUT phenotypes similar to those observed in retinoic acid (RA)-deficient models. Subsequent studies demonstrated that expression of RA receptor alpha was decreased in E13.5 Gen1 mutant ureters and kidneys. Further studies showed that all-trans retinoic acid (ATRA) rescued solitary kidney phenotype and improved ureteric branching in *GEN1*^PB/PB^ mice [32–35]. However, the relationship between *GEN1* and human CAKUT remains unclear. To further investigate the relationship between *GEN1* rare variants and human CAKUT, we analyzed the clinical information of 910 individuals with CAKUT who underwent molecular analysis at our center. We also tested *GEN1* variants in 495 individuals with the non-CAKUT phenotype as an in-house control cohort. Truncated, splice-near, and missense variants with predicted pathogenicity were selected for functional tests in in vitro experiments. Based on the variant frequency in CAKUT individuals, and functional studies in vitro, point-mutant mice were constructed to further verify the CAKUT phenotype identified in human individuals.

## Materials and methods

### CAKUT cohort

Patients diagnosed with CAKUT based on kidney imaging studies before 18 years of age were recruited from the Department of Nephrology and Department of Urology, Children’s Hospital, Fudan University. Data on demographics, genetic sequences, clinical information, renal phenotypes, and extrarenal phenotypes were collected for further analysis. CAKUT mainly includes congenital renal abnormalities (renal hypoplasia/malformation, as well as renal agenesis, renal ectopia, renal malrotation, horseshoe kidney, etc.); obstructive nephropathy (ureteropelvic junction obstruction, ureterovesical junction obstruction, and pelvic ureteric junction obstruction with vesico-ureteric reflux); vesicoureteral reflux and reflux nephropathy; bladder dysfunction (detrusor overactivity, voiding dysfunction, severe bladder-rectal dysfunction, and neurogenic bladder). CAKUT was diagnosed by pediatric nephrologists or urologists based on the relevant clinical signs and imaging findings, mainly including ultrasound, MRI, and urination cystourethrogram. Participants with duplicate or missing personal information, those with uncertain diagnoses, and those diagnosed with ciliary diseases were excluded. We enrolled 910 patients with CAKUT in this study. Among them, DNA samples from 400 children with CAKUT underwent Sanger sequencing of *GEN1* between 2011 and 2013. Samples from individuals with *GEN1* variants were further identified using whole-exome sequencing (WES) to exclude other genes known to cause CAKUT. Samples from the other 510 patients underwent WES between 2014 and 2022, and some also underwent microarray-based copy number variation analysis. DNA was extracted from the blood cells of all subjects.

### Control cohort

We considered the concealed phenotype of CAKUT, gnomAD, as a control. We also enrolled 495 patients with normal urologic ultrasound findings and no previous history of urinary tract infection as an in-house control. This cohort included 247 individuals who underwent Sanger sequencing for *GEN1* between 2011 and 2013 and 248 individuals who underwent WES between 2014 and 2022. The diagnoses of the 495 non-CAKUT patients included 13 nephronophthisis, 159 nephrotic syndrome/proteinuria, 271 glomerular nephritis, and 52 tubular diseases.

### Sanger sequencing and variant analysis

Peripheral blood (2 ml) of all subjects was collected in EDTA anticoagulant tubes and stored at − 20 °C. A QIAGEN DNA Mini Kit (cat. 51,304 and 51,306) was used for DNA extraction. The PCR products were purified, alcoholized, and sequenced from 2011 to 2013. The original data were read and analyzed using Seqencher (version 5.1) software. The standard *GEN1* sequence was downloaded from the Ensemble database (NM_182625; NP_872431). Variants were checked in the population in the Ensemble, NCBI, and gnomAD databases, and were further analyzed using Alamut-HT (Interactive BioSoftware, Rouen, France), which integrates multiple functional prediction databases (Polyphen2, SIFT, and Mutation Taster) for comprehensive analysis. Segregation analysis was performed if parental DNA was available. All the mutation sites were verified in forward and reverse. Subsequently, we performed a WES analysis on individuals whose GEN1 variants had been identified by sanger sequencing to determine the presence of other known genes that cause CAKUT.

### Whole-exome sequencing, next generation sequencing, and variant analysis

Between 2014 and 2022, samples were sent for WES, and if possible, next generation sequencing (NGS). The protocols were described previously [36]. Variants (missense, stop-gain, and splice site) were analyzed using at least four predictors (Polyphen2, Sift, MutationTaster, Grantham score, and Align-GVGD).

### Selection of variants tested in vitro experiments and in a mouse model

The routine of this study is outlined in Figure S2. In vitro, 10 variants (eight CAKUT, two non-CAKUT) were selected to verify mutant protein stability. These eight CAKUT variants were preliminarily predicted through the website(http://genetics.bwh.harvard.edu/pph2/), and the results showed that these seven mutation points involved likely affect protein stability. The two sites in the non-CAKUT group were used as controls and truncated mutations significantly alter protein structure, so the three sites were selected for both protein stability experiments and enzyme-related experiments. According to the distribution of each variant site on the GEN1 protein in Figure S3, Gen1 (p.T105R), Gen1 (p.D149N), Gen1 (p.R401X,508), and Gen1 (p.A105R, I577T) were selected as they located in the domains that related to the enzymatic activity. Gen1 (p.I577T) was selected as it was contained in the variant Gen1 (p.A105R, I577T).Three human variants, Gen1 (p.T105R), Gen1 (p.R401X,508), and GEN1 (c.1071 + 3(IVS10) A > G), were chosen for point-mutant mouse model construction (Gen1 (p.T105R), Gen1 (p.R400X), and Gen1 (c.1068 + 3 A > G)). Gen1 (p.T105R) was selected based on its highest frequency (4/26) among the patients with CAKUT and its associated significant functional impairment detected in the in vitro experiments. Gen1 (p.R400X) was selected based on its high frequency (3/26) in the patients with CAKUT and for having the most severe functional impairment detected in the in vitro experiments. Given the abnormal formation of exon10 detected in the in vitro experiments, Gen1 (c.1068 + 3 A > G) was also selected. The point-mutant mice constructed in this study were all C57BL/6J. The temperature was maintained at 18–22 ºC, the humidity at 50–60%, and the light and dark cycle was 12 h:12 h.

The C57BL/6 mouse strain (Jackson Laboratory, Bar Harbor, ME; #001800) was used to edit *Gen1* using CRISPR-Cas9 technology [37]. The procedure for constructing a point-mutant mice is detailed in Supplemental project 1–3. For genotype identification, a small amount of mouse tail tissue was placed in an Eppendorf tube with SNET lysate (200 µL) and 1/50 proteinase K (B600169-0002, 20 mg/mL, 2 µL), DNA was extracted through overnight lysis at 55 °C, and amplification was performed using KOD Dyemix (TOROIVD, KAO-201). The primers used in this procedure are listed in Table S3. PCR products were sent to Sangon (Shanghai, China) for Sanger sequencing; the sequencing and amplification primers used were the same as those described above.

**Table 1:** Clinical information of patients with *GEN1* variants

Subject number	Nucleotide changes	Amino acid changes	Gender	Age of diagnosis	Renal manifestations	Extrarenal manifestations	Last investigation age / GFR category	Variation segregation	gnomAD*
C1	c.2642 A > C	p.H881P	F	2y	BL duplex kidney, R ureteral ectopia, ureteral cyst, VUR (R V)	None	6y / G1	unknown	0/7/241,030
C2	c.2633T > C	p.L878P	F	1y	VUR (R IV, L IV)	None	4y9m / G1	unknown	0/5/246,044
C3	c.656 A > G	p.K219R	M	10y	L RHD, VUR (R II, L III)	None	9 m / G1	unknown	0/2/249,978
C4	c.2527 C > T	p.P843S	M	1y	horseshoe kidney, renal malrotation, VUR (R IV, L IV)	Microcephaly, delayed language development, atrial septal defect, umbilical hernia, short stature	3y4m / G1	unknown	0/7/250,554
C5	c.181T > A	p.S61T	F	5 m	VUR (L III)	None	1y4m / G1	unknown	0/42/275,818
C6	c.1201 C > T	p.R401X,508	M	8y	R RHD, VUR (R IV)	None	7y5m / G1	unknown	0/12/273,278
C7	c.824G > A	p.R275H	F	6y	VUR (L III)	None	15y2m / G1	unknown	1/48/280,456
C8	c.590 C > T	p.A197V	M	2y	L RHD, VUR (R V, L V)	None	1y9m / G3b	unknown	Not reported
C9	c.761T > C	p.V254A	M	1y	VUR (L III), PUV	Hydrocele	1y / G1	unknown	Not reported
C10	c.974G > T	p.G325V	F	2.5y	VUR (R IV, L IV)	None	4y4m / G1	unknown	0/7/279,952
C11	c.445G > A	p.D149N	M	4y	VUR (L IV)	None	3y7m / G1	unknown	0/12/251,480
C12	c.1356 C > G	p.I452M	M	3.5y	L RHD, VUR (R IV, L IV)	None	5y5m / G2	unknown	0/5/249,936
C13	c.181T > A	p.S61T	M	12y	BL RHD, VUR (L II)	Short stature	17y / G3b	father	0/42/275,818
C14	c.2327 A > G	p.D776G	F	10.5y	RHD(BL)	Dilated cardiomyopathy, short stature	12y4m / G5	mother	0/5/250,374
C15	c.1201 C > T	p.R401X,508	M	15.5y	BL RHD, VUR (L IV)	None	15y5m / G4	unknown	0/12/273,278
C16	c.1071 + 3(IVS10) A > G	/	F	3.5y	VUR (R II)	Both knees valgus	4y / G5	Paternal	Not reported
C17	c.1106 A > T	p.H369L	M	5y	VUR(R IV), RHD	None	10y / G2	Maternal	Not reported
C18	c.314 C > G	p.T105R	M	2 m	RHD	None	7y6m / G3a	unknown	0/21/279,086
C19	c.1609T > C	p.C537R	F	9y	RHD, VUR(RIII)	None	12y / G1	mother	0/7/250,878
C20	c.1201 C > T	p.R401X,508	F	6y	UVJO	None	11y5m / G1	mother	0/12/273,278
C21	c.314 C > G + 1730T > C	p.T105R + p.I577T	M	before birth	PUJO	None	2y7m / G2	respectively from parents	/
C22	c.1609T > C	p.C537R	M	before birth	Congenital ureterovesical opening stenosis	None	4y3m / G2	unknown	0/7/250,878
C23	c.314 C > G	p.T105R	F	1y	VUR(RIII)	None	10y5m / G1	father	0/21/279,086
C24	c.1730T > C	p.I577T	M	before birth	PUJO(BL)	None	4y7m / G1	mother	0/2/250,480
C25	c.58 C > T + 181T > A	p.R20C + p. S61T	F	3y	VUR(LIV RIV)	None	4y6m / G2	respectively from parents	/
C26	c.314 C > G	p.T105R	F	/	solitary kidney (R absence)	None	/	unknown	0/21/279,086
N1	c.1657 A > G	p.I553V	M	2y	Microscopic polyangiitis	Hematuria, NS	1y10m / G3a	father	Not reported
N2	c.2116T > C	p.S706P	F	11y	Growth and development retardation, brain atrophy	Alport syndrome	1y6m / G5	father	0/2/250,596

Abbreviation: BL, bilateral; CKD, chronic kidney disease; C1-26, subject 1-26 with CAKUT phenotype; F, female; L, left; m, month; M, male; MCDK, multicyclic dysplastic kidney; NS, nephrotic syndrome; N1-2, subject 1-2 without CAKUT phenotype; PUJO, ureteropelvic junction obstruction; PUV, posterior urethral valve; R, right; RHD, renal hypoplasia (renal dysplasia); UVJO, ureterovesical junction obstruction; VUR, vesicoureteral reflux; y; year. A Allele frequencies in gnomAD are indicated as homozygous/hemizygous (if applicable)/heterozygous/total alleles. “/” Data deficient. The “unknown” in variation segregation indicated that samples from the participants' parents were not collected, thus the genetic origin could not be determined.

### Mice CAKUT phenotype observation

Newborn mice were euthanized via CO_2_. The abdominal cavity was dissected, and the gastrointestinal tract and liver were peeled apart. The bladder was carefully exposed, and a VUR experiment was performed as described in our previous work [38]. The upper body was cut off, and the lower body was placed under a posture microscope to carefully dissect and photograph the urinary system. All observed phenotypes were recorded.

### Quantitative real-time PCR

Total RNA was extracted from E11.5 mice, using TRIzol reagent (Life Technologies, USA). The RNA concentration and purity were measured using the optical density ratio at 260 nm/280 nm. Each sample (1.0 µg RNA) was reverse transcribed into cDNA using PrimeScript (TaKaRa, China), as per the manufacturer’s instructions. PCR was performed using AceQ qPCR SYBR Green Master Mix (Vazyme, China) and a quantitative real-time (qRT)-PCR system (Agilent Mx3000P, USA). GAPDH was used as the control. All primers were obtained from the NCBI GenBank database and synthesized by Sangon (Shanghai, China). The primers used were as follows: *Gen1*-F:5′-GCCTGGAGTTGGAAAGGAACAAG3′, *Gen1*-R:5′-GGAACACACAGAGCAGTGAACCAC-3′, *Gapdh*-F:5′-TGTTCCTACCCCCAATGTGTCC-3′, and *Gapdh*-R:5′-GGAGTTGCTGTTGAAGTCGCAG-3′. Relative gene expression levels were calculated using the 2^− ΔΔCT^ method, with three samples per group, each replicated at least three times [38].

Further details of the methods for the in vitro experiments are provided in the Supplement methods.

### Statistics

Differences in the *GEN1* variation rate between the CAKUT and non-CAKUT groups were evaluated using Fisher’s exact test. Statistical tests were performed using GraphPad Prism v.8 and GraphPad Software. The statistical significance of CAKUT incidences and *Gen1* expression between C57 WT and point-mutant mice was evaluated using unpaired t-tests. *Gen1* expression analyses were performed at least three times. Statistical significance was defined as *P* < 0.05. A single asterisk indicates *P* < 0.05, whereas double, triple, and quadruple asterisks indicate *P* < 0.005, *P* < 0.0005, and *P* < 0.0001, respectively. Standard deviation (SD) was used for all experiments, except for the migration rate, where the standard error of the mean (SEM) was used instead.

## Results

### *GEN1* rare variants in CAKUT subjects

In this study, 910 patients with CAKUT were included; of these, 400 children from 2011 to 2013 underwent Sanger sequencing, and 10 of 400 individuals (2.5%) were found to carry rare *GEN1* variants (nine were heterozygous and one was compound heterozygous) without pathogenic variants identified in known genes causing CAKUT. These 10 children who were detected with the *GEN1* variant by Sanger were also scheduled for WES to further rule out the presence of other known CAKUT pathogenic gene variants. WES analysis was performed on the other 510 children between 2014 and 2022, and 16 of the 510 (3.1%) patients carried rare heterozygous *GEN1* variants. Detailed genetic and clinical information of these 26 patients with CAKUT and *GEN1* variants is listed in Table1. We identified one stop-gain variant in three individuals (c.1201 C > T, p. R401X,508, 3/26), one splice site variant in one individual (c.1071 + 3 A > G, 1/26) and 17 missense variants in 22 individuals (22/26). Among these 26 individuals, 12 were female and 14 were male.

The CAKUT phenotypes observed in these 26 individuals included unilateral renal agenesis, renal dysplasia, multicyclic dysplastic kidney disease, ureteropelvic junction obstruction (PUJO), ureterovesical junction obstruction (UVJO), posterior urethral valves (PUV), and VUR (Table 1). Among these presentations, VUR was most common, seen in 19 of 26 patients (61.5%). Nine had renal dysplasia, and one had a solitary kidney. Extrarenal phenotypes were observed in five patients (5/26, 19.2%).

We also analyzed *GEN1* rare variants based on the same criteria in the non-CAKUT group as an in-house control cohort. Sanger sequencing of *GEN1* was performed in 247 patients between 2011 and 2013, and no *GEN1* variants were found. The remaining 248 patients between 2014 and 2022 underwent WES analysis, of which two patients with *GEN1* heterozygous missense variants were identified (Table1). However, these two variants were not found in the CAKUT group. Our results indicate that *GEN1* variants were present in 26 patients among 910 patients with CAKUT, and two *GEN1* variants were present in 495 non-CAKUT subjects. Further analysis showed that the *GEN1* variant carrier rate in the CAKUT population was significantly higher than that in the control group (*P* = 0.003, **χ**^2^ = 8.662).

### Decreased stability of GEN1 point mutant proteins

**Fig. 1:**
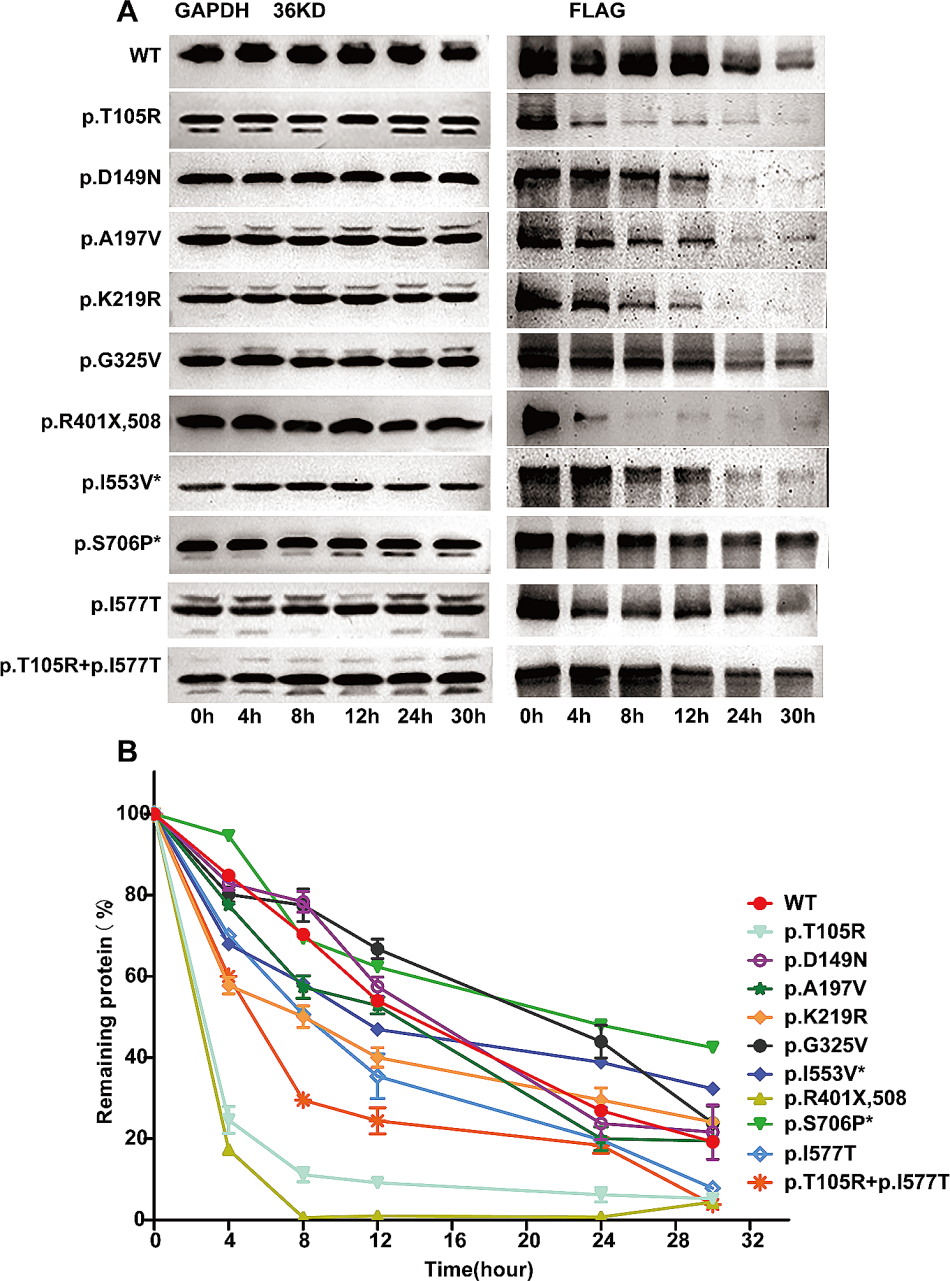
Stability of point-mutated GEN1 proteins. The protein stability was measured by the rate of wild-type and mutant protein degradation under the same conditions, and the stability results of all mutated proteins were evaluated from the baseline results of wild-type proteins. (**A**) Western blot analysis of point-mutant proteins and wild-type GEN1 protein treated with cycloheximide at 0, 4, 8, 12, 24, and 30 h. The grayscale bands represent the amount of protein involved. * sites in the non-CAKUT group. The left side band in Figure A is the inner reference GAPDH, and the right band is the wild-type and mutant protein displayed using the FLAG label. (**B**) Grayscale chart of the western blot analysis. The results are reported as mean ± SEM for three individual experiments. * sites in the non-CAKUT group. Abbreviations: SEM, standard error of the mean

The locations of the mutated amino acid residues in the *GEN1* protein domain are shown in Figure S3. Specific plasmids with subject variants were constructed to explore changes in the stability of the mutant proteins. Since the results predicted that mutant protein stability was impaired, mutant protein stability was tested at eight sites (p.T105R, p.D149N, p.A197V, p.K219R, p.G325V, p. R401X,508, p. I577T, and p. T105R + p. I577T) in the CAKUT group. Additionally, we tested mutant protein stability (p.I553V and p.S706P) in the non-CAKUT group because these two variants were located in an unknown region (UR) of GEN1 and cannot predict the affecting of protein stability. Western blot images of cycloheximide-treated transfected cell proteins suggested that the protein stability changed in six of eight variants in the CAKUT group but did not obviously change in the two variants in the non-CAKUT group (Fig. 1A). Gen1 (c.1201 C > T, p.R401X,508) and Gen1 (c.314 C > G, p.T105R) stability decreased the most, and the residual protein content decreased to 10% at 8 h after cycloheximide treatment. Other mutations with reduced stability included GEN1 (p. T105R + p. I577T), GEN1(c.1106 A > T, p.H369L), and GEN1 (c.656 A > G, p.K219R). Notably, the GEN1 (c.974G > T, p. G325V) protein demonstrated increased stability, and the protein content remained as high as 40% at 24 h. Protein stability did not change significantly in GEN1 (p.D149N) or GEN1 (p.A197V) in the CAKUT group and in GEN1 (p.I553V) and GEN1 (p.S706P) in the non-CAKUT group.

### As HJ resolvases, DNA binding ability and enzymatic hydrolytic capacity were damaged in mutant GEN1 proteins

Electrophoretic mobility shift assay (EMSA) allows for the comparison of changes in the ability of GEN1 to bind to DNA (HJs). EMSA determines the protein-DNA (HJ) binding capacity by separating the protein-DNA conjugate from the DNA (HJ) substrate. Four sites (c.314 C > G, c.445G > A, c.1106 A > T, c.1201 C > T) located in the enzyme activity region, and one site (c.1730T > C) in the UR, constituting one of the compound sites (c.314 C > G + c.1730T > C), were selected to test the enzyme activity (Figure S2). We also tested two sites (c.2116T > C and c.1657 A > G) in the non-CAKUT group because they are located in the UR of GEN1. The different mutant GEN1 proteins had varying degrees of reduced binding capacity to HJ compared to WT GEN1, with GEN1 (p.R401X, 508) having the worst functional damage, followed by GEN1 (p.T105R + p.I577T) and GEN1 (p.T105R). The protein-DNA (HJ) binding capacity change of the two variants in the non-CAKUT group was slightly (Figure S4).

**Fig. 2:**
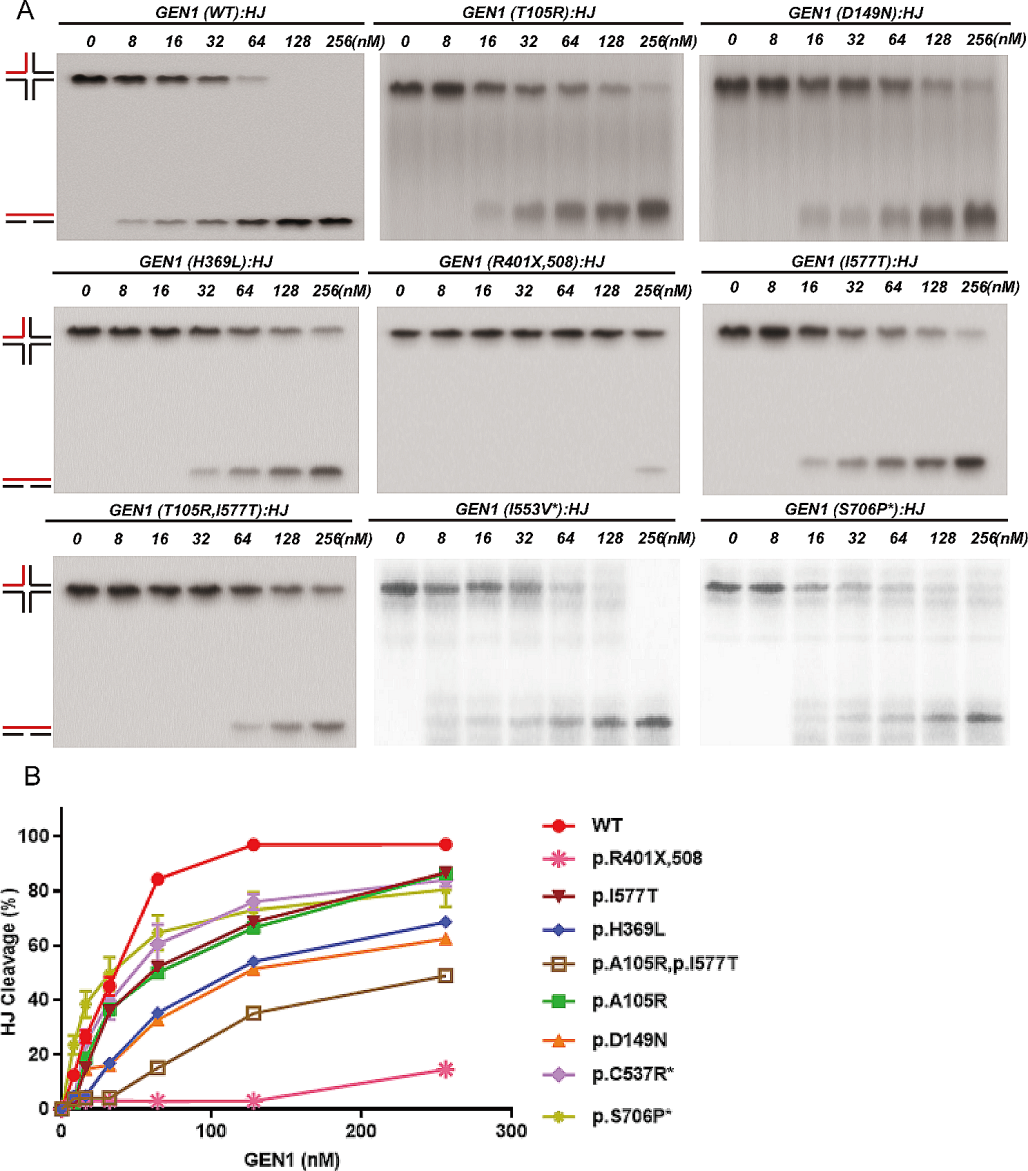
Nuclease activity of GEN1 variant proteins at HJs. As a HJ enzymatic lyase, GEN1 protein’s dissociation ability was partly manifested by the degradation of the equal HJ (substrate) by different concentrations of purified proteins. In this experiment, seven gradient concentrations were applied (0, 8, 16, 32, 64, 128 and 256nM). GEN1 (p.R401X,508) showed the most significant reduction in enzymatic hydrolytic capacity. The other five mutant proteins [GEN1 (p.T105R), GEN1 (p.D149N), GEN1 (p.H369L), GEN1 (p.I577T), and GEN1 (p. T105R + p.I577T)] impaired the enzymatic hydrolysis of HJ. The impaired enzymatic hydrolytic capacity in the control group variants was less significant than that in CAKUT group. (**A**) Grayscale statistics for the above grayscale map, reflecting the comparison of the ability of wild-type and mutant GEN1 protein to degrade HJ. All results of all mutated proteins were evaluated from the baseline results of wild-type proteins. (**B**) Grayscale statistical chart. The results are reported as mean ± SD for three individual experiments. * sites in the non-CAKUT group. Abbreviations: EMSA, electrophoretic mobility shift assay; HJ, Holliday junction; WT, wild type

**Fig. 3:**
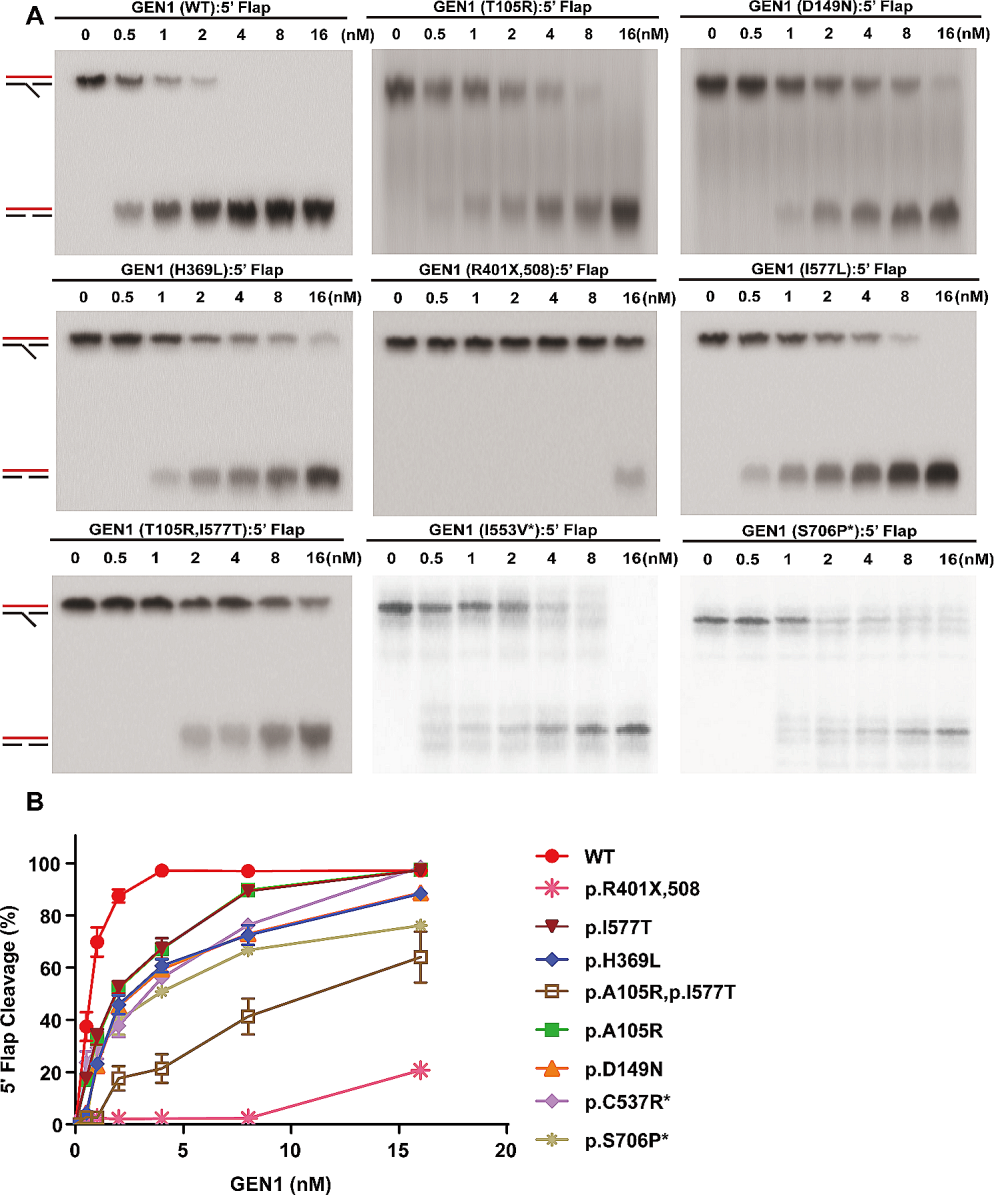
Nuclease activity of GEN1 variant proteins at 5’ flap DNA. As a HJ enzymatic lyase, GEN1 protein’s dissociation ability was partly manifested by the degradation of the equal 5’ flap (substrate) by different concentrations of purified proteins. In this experiment, seven gradient concentrations were applied (0, 0.5, 1, 2, 4, 8 and 16nM). GEN1 (p.R401X,508) showed the most significant reduction in enzymatic hydrolytic capacity. The other five mutant proteins [GEN1 (p.T105R), GEN1 (p.D149N), GEN1 (p.H369L), GEN1 (p.I577T), and GEN1 (p. T105R + p.I577T)] impaired the enzymatic hydrolysis of 5’ flap. The impaired enzymatic hydrolytic capacity in the control group variants was less significant than that in CAKUT group. (**A**) EMSA of GEN1-5’ flap complexes. 5’ flap DNA was hydrolyzed by WT and mutant GEN1 proteases. The upper band of each plot represents undigested 5’ flap DNA, and the lower band represents enzymatically hydrolyzed 5’ flap DNA. The band concentration represents 5’ flap concentration. (**B**) Grayscale statistical chart. The results are reported as mean ± SD for three individual experiments. * sites in the non-CAKUT group. Abbreviations: EMSA, electrophoretic mobility shift assay; HJ, Holliday junction; SD, standard deviation; WT, wild type

EMSA can also determine the enzymatic hydrolytic capacity by separating the substrates and products for enzymatic hydrolysis. The enzymatic hydrolysis of HJ and 5` flap capabilities of the GEN1 proteins are shown in Figs. 2 and 3. GEN1 (p.R401X,508) showed the most significant reduction in enzymatic hydrolytic capacity, which was 20% of that of WT GEN1. The other five mutant proteins (GEN1 (p.T105R), GEN1 (p.D149N), GEN1 (p.H369L), GEN1 (p.I577T), and GEN1 (p. T105R + p.I577T)) impaired the enzymatic hydrolysis of both HJ and 5` flaps. Notably, the reduction in enzymatic activity of GEN1 (p.T105R + p.I577T) was more dramatic than that of GEN1 (p.T105R) or GEN1 (p.I577T). The impaired enzymatic hydrolytic capacity in the control group variants was less significant than that in the CAKUT group variants.

### Splice site GEN1 (c.1071 + 3(IVS10) A > G) influenced Exon10 formation

**Fig. 4:**
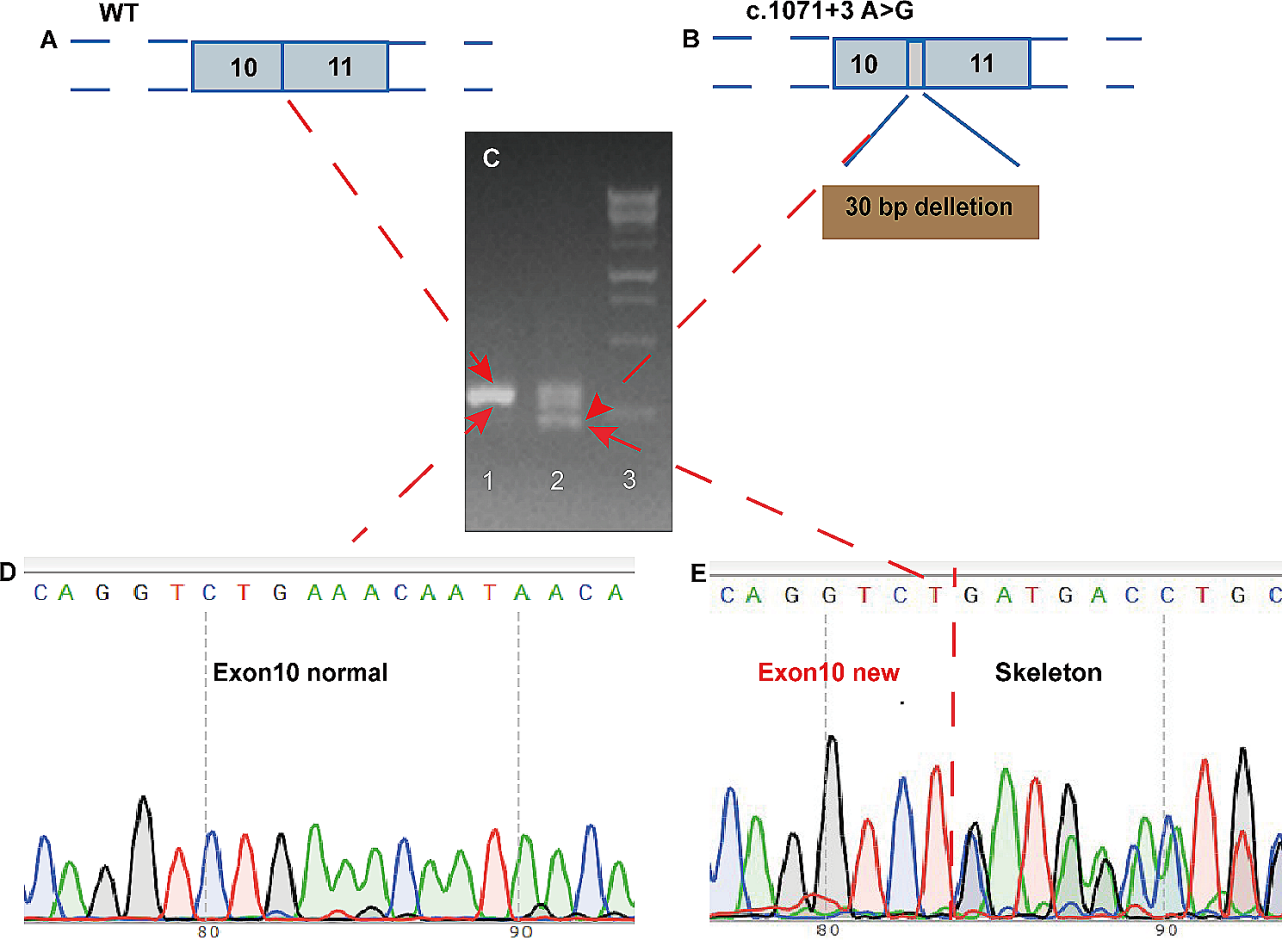
PCR product electrophoresis and sequencing results. PCR substances of shear site variation and wild-type plasmid transcripts were used to identify the difference between the two transcripts. Shear site variation formed shorter exons 10. The sequencing results further confirmed the missing 30 bp sequence. (**A, B**) Simple schematic diagrams of mRNA formed by WT and Gen1 (c.1071 + 3(IVS10) A > G) mutant proteins. (**C**) Electrophoresis of PCR products. Lane 1 is the wild type, lane 2 is the shear variant, and lane 3 is the marker. (**D, E**) Sanger sequencing of PCR products. Abbreviations: MT, mutant type; WT, wild type

We identified a heterozygous splice site variant in one individual with CAKUT located in intron 10 of GEN1 (c.1071 + 3 A > G), which has not been reported in a public database (in either 1000 Genomes or The Genome Aggregation Database); hazard prediction analysis indicated that GEN1 (c.1071 + 3 A > G) was damaging. To further explore any effects on splicing function, the WT (TCS134-1: pSPL3b[minigene] SV40>{hGEN1(E9–10 + intron)} and MT (TCS134-2: pSPL3b[minigene]-SV40>{hGEN1(E9–10 + intron)} plasmids were subjected to in vitro experiments. By investigating mRNA splicing abnormalities with electrophoresis, the product formed by transfection with the WT plasmid provided a single band (Fig. 4), while the product formed by transfection with the MT plasmid was separated into multiple bands; one band was smaller than the WT electrophoretic band indicated new product generation. Sanger sequencing of cDNA revealed that the c.1071 + 3 A > G mutation was associated with a new, shorter exon 10 representing a deletion of 30 nucleotides in exon 10 compared with the WT. In addition, other bands were collected for sequencing analysis, and no other abnormal shearing was found. The sequencing file has been added in the Supplement sequence files.

### All three point-mutant strains exhibited CAKUT phenotypes

To further explore the CAKUT pathogenicity of the *GEN1* mutant locus in patients, we constructed three strains of point-mutant mice, GEN1 (p.T105R), GEN1 (p.R401X,508), and GEN1 (c.1071 + 3 A > G), based on the frequency of variants in the CAKUT group and the effects of the function of GEN1 protein in the in vitro experiments described above. Gen1 (p.T105R), Gen1 (p.R400X), and Gen1 (c.1068 + 3 A > G) mutant mice were constructed using CRISPR/case9 technology.

**Fig. 5:**
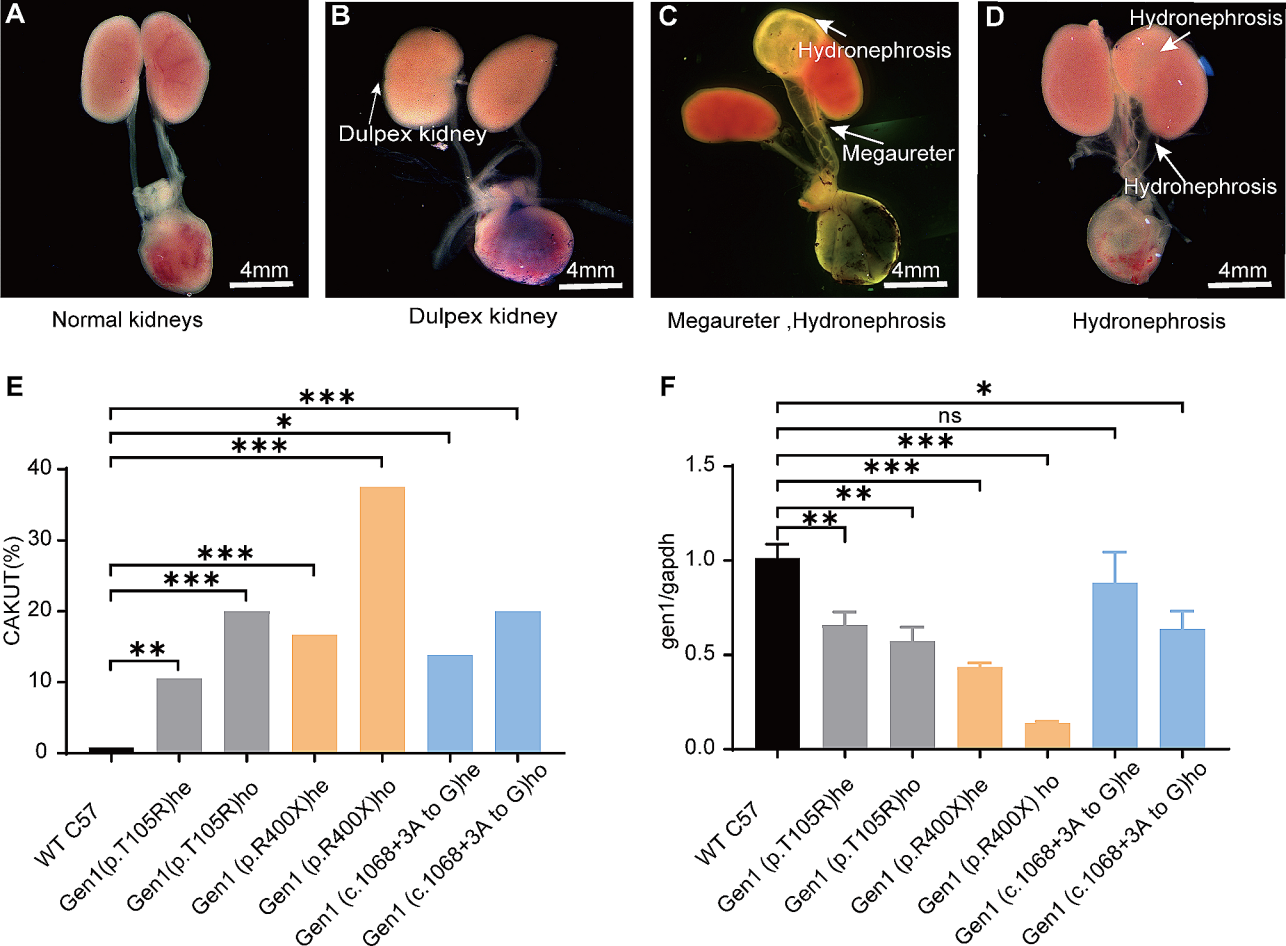
Phenotypic and gene expression analysis in three point-mutant mice strains in postnatal days. (**A**) Normal kidneys. (**B**) Duplex kidney. (**C**) Hydronephrosis, duplex kidney and megaureter. (**D**) Hydronephrosis. (**E**) CAKUT frequency in WT (C57), Gen1 (p.T105R) HE (2/19, 10.52%), Gen1 (p.T105R) HO (1/5, 20%), Gen1 (p.R400X) HE (10/62, 16.13%), Gen1 (p.R400X) HO (3/8, 37.5%), Gen1 (c.1068 + 3 A to G) HE (4/29, 13.8%), and Gen1 (c.1068 + 3 A to G) HO (1/5, 20%) mice. Sample size per group is represented in the image. (**F**) *Gen1* expression in FVB wild-type and point mutant mice of various strains. All analyses of variance were compared to the WT group.* *P* < 0.05, ** *P* < 0.01. The results are reported as mean ± SEM for at least three individual experiments. Abbreviations: HE, heterozygous; HO, homozygous; SEM, standard error of the mean; WT, wild type

Gen1 (p.R400X) showed the most significant decrease in *Gen1* expression in the whole body of each strain of neonatal mutant mice, with heterozygous and homozygous *GEN1* expression decreasing to approximately 40% and 20%, respectively, compared to WT (C57) mice [35]. The two residual strains showed fewer significant differences in *GEN1* expression compared to the WT mice (Fig. 5F**).**

The renal phenotypes of each genotype showed that all point-mutant mice exhibited the CAKUT phenotype. Furthermore, similar to *Gen1*^*PB/PB*^ mice [35], CAKUT incidence was significantly correlated with decreased *Gen1* expression. In the Gen1 (p.T105R) mutant mice, the CAKUT phenotype was identified in 10.52% of heterozygous (2/19) and 20% of homozygous (1/5) mice (Fig. 4E). In Gen1 (p.R400X) mutant mice, the CAKUT phenotype was identified in 16.13% of heterozygous mice (10/62) and 37.5% of homozygous mice (3/8). In Gen1 (c.1068 + 3 A > G) mutant mice, the CAKUT phenotype was identified in 13.8% of the heterozygous mice (4/29) and 20% of the homozygous mice (1/5). The presence of the CAKUT phenotype in these three mouse strains suggests that the selected GEN1 variant sites could cause CAKUT.

## Discussion

We explored the correlation between *GEN1* rare variants and human CAKUT. The incidence of rare *GEN1* variants was significantly higher in the CAKUT group (26/ 910 variants in the CAKUT group vs 2/495 variants in the control group, P = 0.003). Gen1 (p.T105R) showed the highest frequency (4/26) followed by Gen1 (p.R400X,508) (3/26) among patients with CAKUT. Further protein stability experiments showed that mutant protein stability changed in six of eight variants in the CAKUT group but not in the control group; Gen1 (p.R401X,508) and Gen1 (p.T105R) showed the most decreased stability. For DNA binding ability, the stop-gain variant Gen1 (p.R401X, 508) showed the greatest damage. The ability to enzymatically hydrolyze HJ and 5’ flap in six variants of the CAKUT group was tested using EMSA, revealing that this ability was damaged to varying degrees. When testing mutant enzymatic activity, the stop-gain variant Gen1 (p.R401X, 508) and the compound heterozygous variant (c.314 C > G (p.A105R) + c.1730T > C (p.I577T)) showed the greatest damage to GEN1 WT protein function. Additionally, the splice site variant Gen1 (c.1071 + 3 A > G) produced a shorter exon 10. Furthermore, taking the variants frequency and functional studies together, by generating mouse models of three *Gen1* mutations (Gen1 (p.T105R), Gen1 (p.R400X), and Gen1 (c.1068 + 3 A > G)) identified in the CAKUT group, the CAKUT phenotypes were replicated, confirming the importance of *Gen1* in renal development. Therefore, our study indicates that *GEN1* is a risk factor of human CAKUT.

The etiology of CAKUT is complex and involves genetic, epigenetic, and environmental factors. Although genetic factors have been studied recently, only 50 reported genes related to CAKUT in humans have been isolated [39]. However, these genes only account for no more than 20% of the cases of CAKUT. In our previous studies, *Gen1* PB insertion mice developed phenotypes consistent with human CAKUT phenotypes; *Gen1*^*PB/PB*^ mice demonstrated reduced Gen1 expression (down to 15%) and a CAKUT incidence of 50%, while *Gen1*^*PB/+*^ mice demonstrated a less severe reduction in Gen1 expression (down to 60%) and a CAKUT incidence of 20% [35]. GEN1 is a HJ-dissociating enzyme first identified in 2008 containing 908 amino acids and with a molecular mass of 110 kDa [24]. The gene encodes a member of the Rad2/XPG nuclease family characterized by an N-terminal and internal pigmented dry dermatophyte group G nuclease structural domain, followed by a helix-hairpin-helix structural domain and disordered C-terminal structural domains [40]. The protein encoded by this gene is involved in HJ resolution [29, 41]. The GEN1 dimer is the functional form of this enzyme that performs HJ cleavage [42]. RNA sequencing suggests that the expression of Raldh2, a key enzyme in RA synthesis, was reduced in *Gen1*^*PB/PB*^ mice and that the CAKUT phenotype observed in *GEN1* PB insertion mice was similar to that in RA-deficient mouse models [34]. Furthermore, application of ATRA can rescue the solitary kidney phenotype and improve ureteric branching. In addition, the exploration of the mechanism in *GEN1* PB insertion mice further suggested that GEN1 interacts with SIX1 and enhances the transcriptional activity of SIX1/EYA1, which is a key regulatory complex of GDNF morphogenes. The impair expression of *Grem1* and *Gdnf*, resulted in excessive ureteral bud formation and defective ureteral bud branching during early renal development [32].

CAKUT is characterized by its heterogeneity in clinical and genetic conditions, which is mainly demonstrated by its diverse phenotypes and incomplete phenotypic penetration and which make CAKUT etiology studies difficult to conduct [10, 43, 44]. Family members with the same genetic defects can even have urinary phenotypes ranging from asymptomatic to advanced renal failure [45]. For example, among the harmful variants or deletions identified in TOM1L2, the majority of patients inherited mutations from their asymptomatic parents, except for a few de novo variants [46], which can be considered a concrete manifestation of incomplete explicit characteristics. In addition to the dominant role of genetic factors, environmental factors and epigenetic factors (such as DNA methylation) are involved in CAKUT pathogenesis [39], which should be explored in the future.

In a study exploring the correlation between *ROBO2* variations and human CAKUT, only 1/6 of the mutations were de novo [47]. *Robo2*^*PB/+*^ mice showed an approximately 15% CAKUT incidence [38]. Only 34.37% (11/32) of children with *PAX2* mutations have the CAKUT phenotype [48], and ∼ 6% of patients with *PAX2* variations do not show any renal phenotype [49]. Furthermore, 14% of *Pax2*^*+/−*^ mice developed the CAKUT phenotype [50]. The phenotypes of the same defective gene in mice are diverse and usually include VUR, hydronephrosis, duplicated kidneys, renal dysplasia, and giant ureters. Our results also suggested that some *Gen1* variants (c.181T > A, c.2327 A > G, c.1071 + 3 A > G, c.1106 A > T, c.1609T > C, c.1201 C > T, c.314 C > G, and c.1730T > C) in the CAKUT group were from asymptomatic fathers or mothers, and mutant mice with CAKUT incidences ranging from 10 to 38%, which indicates incomplete penetrance. In the non-CAKUT group, the two sites investigated in our study were in unknown functional domains, and in vitro validation confirmed that these variants did not affect GEN1 function, indicating that variants in different regions might indicate different pathogenicity.

Our GEN1 functional study also indicated that different variants had different degrees of functional impact. Truncated variants had the greatest effect, and altered splicing sites also affected function. Compound heterozygous effects were more obvious, which explains the diversity of the phenotypes to a certain extent. Additionally, the two *Gen1* variants in the non-CAKUT group showed no obvious functional impairment compared to the variants in the CAKUT group, indicating that different variations in the same gene can lead to varying degrees of functional impairment.

Our study suggests that the association between CAKUT and *GEN1* variants is heterogeneous. The constructed mice exhibited CAKUT phenotypes, reflecting the CAKUT pathogenicity of these *GEN1* variants. However, our study had some limitations. First, this was a single-center study. Therefore, we need to explore *GEN1* carriers of different ethnicities. Second, the pathogenicity of different sites requires functional verification of more sites and the construction of additional point-mutant mice. To further clarify the causative factors of *GEN1* variants in human cases of CAKUT, *GEN1* variant analysis in different ethnicities, further site function validation and the construction of point-mutant mice are required.

### Electronic supplementary material

Below is the link to the electronic supplementary material.

Supplementary Material 1

Supplementary Material 2

Supplementary Material 3

Supplementary Material 4

Supplementary Material 5

Supplementary Material 6

## Data Availability

The human GEN1 full-length protein sequence can be found in GenBank: NM_001130009.3.
