# Supplementary material for: GEN1 as a risk factor for human congenital anomalies of the kidney and urinary tract
Source: Hum Genomics. 2024 Apr 24;18:41. doi: 10.1186/s40246-024-00606-8 (PMC11041010; doi:10.1186/s40246-024-00606-8)
Supplement: Supplementary file 1 — Supplementary Material 1 [file 40246_2024_606_MOESM1_ESM.docx]

**1. cDNA cloning**

**2. Cell culture and transient transfection**

**3. Stability detection of point-mutant proteins**

**4. Western blot analysis**

**5. Point mutation protein purification**

**6. Protease activity validation of mutant proteins**

**7. Electrophoretic mobility shift assay**

**8. Verification of mRNA splicing abnormalities**

**1. cDNA cloning**

The GEN1 cDNA ([CCDS1691.1](https://www.ncbi.nlm.nih.gov/CCDS/CcdsBrowse.cgi?REQUEST=CCDS&GO=MainBrowse&DATA=CCDS1691.1)) plasmid was obtained from Sino Biological (HG21694-ANG PCMV3-GFPSpark-GEN1-t2). Mutagenesis was performed using the KOD-Plus-Mutagenesis Kit SMK-101 (TOYOBO Bio-Technology) to generate clones with the *GEN1* mutations identified in patients. Each constructed plasmid was sequenced to verify the correct frame and proper sequence of any linkers introduced during cloning. The mutation sites, mutant primer sequences, and mutant plasmid sequencing primers are listed in **Table S1**. The GEN1([CCDS1691.1](https://www.ncbi.nlm.nih.gov/CCDS/CcdsBrowse.cgi?REQUEST=CCDS&GO=MainBrowse&DATA=CCDS1691.1))-FLAG plasmid was constructed by JustScience (Shanghai, China). The inserted gene sequence and plasmid structure diagram are shown in **Figure S1**. Mutagenesis was performed using KOD-Plus-Mutagenesis Kit SMK-101, and all constructs were sequenced to confirm the correct sequence. The minigene splicing plasmids WT (TCS134-1:pSPL3b[minigene]-SV40>{hGEN1 (E9~10+ intron)}) and mutant type (MT) (TCS134-2: pSPL3b [minigene]-SV40> {hGEN1(E9~10+ intron)}) were constructed by Suzhou Haixing Biological Technology. Sequencing was performed before all the plasmids were applied.

**2. Cell culture and transient transfection**

cDNA clone transfection experiments were performed using human embryonic kidney (HEK293) cells purchased from the American Type Culture Collection Bioresource Center. HEK293T cells were cultured in high-glucose DMEM (Gibco) supplemented with 10% FBS (Gibco) and 1% penicillin (Gibco). At 37°C, 5% CO_2_, cells were transiently transfected using Lipofectamine 2000 transfection reagent (Thermo Fisher Scientific) and OptiMEM (Thermo Fisher) at 60–70% confluence according to the manufacturer's instructions[^37^](#_ENREF_37). Cycloheximide (CHX, final concentration 100 ng/ml) was added 48 h after plasmid transfection, and the protein was extracted at the corresponding time point after the addition of CHX.

**3. Stability detection of point-mutant proteins**

All GEN1-FLAG WT or site-mutant plasmids were respectively transfected into HEK293T cells by Lipofectamine 2000 (Thermo Fisher) for 48 h. CHX (actinomycin, final concentration 100 μg/ml) was added, and total cell protein was extracted at 0, 4, 8, 12, 24, 30, and 36 h. Western blot analysis verified the degradation curves of FLAG-tagged proteins at different time points, which reflected the changing trend in the stability of mutant proteins at each point compared with WT proteins. All experimental procedures were performed at least three times for each mutant protein, and at least three valid experimental results were included in the final analysis.

**4. Western blot analysis**

Radio immunoprecipitation assay lysis buffer (with phenylmethylsulfonyl fluoride) was used to lyse the transfected CHX-treated HEK293T cells with the addition of 1/3 the volume of 4 × loading buffer. SDS-PAGE was performed using an equal amount of protein, and the PVDF membrane was transferred after electrophoresis (300 mA, 120 min). The PVDF membrane was blocked with 5% skim milk for 1–2 h. The primary antibodies used are as follows: GAPDH (Cell signaling, 5174S,1:1000) and DYKDDDDK Tag (Invitrogen, MA1-91878-HRP, 1:1000). The secondary antibodies used are as follows: HRP-labeled Goat Anti-Rabbit IgG(H+L) (Biyuntian, A0208) and HRP-labeled Goat Anti-Mouse IgG(H+L) (Biyuntian, A0216). The primary antibody was diluted with a primary antibody diluent (Biyuntian, 1:1000), and the secondary antibody was diluted with 1 × Tris-buffered saline with 0.1% Tween® 20 detergent (TBST, 1:1000). The membrane was incubated with the primary antibody at 4°C overnight and the secondary antibody at room temperature on a shaker for 4–6 h. Following incubation with the secondary antibody, the PVDF membrane was washed with 1 × TBST for 10 min three times. Labelled proteins were detected by applying BeyoECL Star ([BeyoECL Plus](https://www.beyotime.com/product/P0018S.htm), P0018S) for 1–2 min. The PVDF membrane was then removed and placed in a Western Blot developer (Tanon 1600) for photography. ImageJ software was used for protein band quantification to assist with grayscale analysis (<https://cnij.imjoy.io/>).

**5. Point mutation protein purification**

The GEN1-HIS WT and mutant plasmids were constructed based on the WT and mutant plasmid by seamless cloning kit (MedChemExpress, HY-K1041). All plasmids were transformed into receptive *E. coli*, coated onto Luria-Bertani (LB) agar plates (Amp+), and then cultured in a 37°C incubator overnight (approximately 12 h). Monoclones were then picked, removed, proliferated, and sequenced to determine plasmid construction. Sequenced strains were inoculated into 10 mL LB culture medium (Amp+ 100 μg/ml) in a 37°C shaker overnight. The overnight bacterial solution was transferred to 200 mL LB liquid medium (Amp+ 100 μg/ml), the initial OD600 was measured, and the cultures were incubated at 37°C, with 180 rpm shaking, to the logarithmic growth phase (OD600 0.6–0.8). After adding isopropyl ß-D-1-thiogalactopyranosid to induce protein expression, the cultures were incubated for 6 h (30°C, 180 rpm). The induced bacterial solution was centrifuged at 4°C and 8000 rpm for 10 min, the supernatant was discarded, and the bacterial pellet was collected. The bacterial pellet was resuspended by adding lysate (20 mM Tris-HCl, 150 mM NaC1, 10% glycerol, pH 8.0) and using a cell ultrasonic disruptor with an amplitude of 35%, a frequency of 3 s at 5 s stop, and an effective ultrasonic time of 30 min. The resulting liquid was centrifuged at 4°C and 10,000 rpm for 30 min. The supernatant was collected after centrifugation and filtered through a 0.45 μm membrane. After a ddH_2_0 flush of the Ni-NTA affinity resin, lysate was added to equilibrate the resin. The filtered supernatant was added to the column, and the effluent was collected. This operation was repeated three times to collect the effluent. Then, 20 mL of lysate-equilibrating resin was added to each pre-cooled wash column, followed by 100 mL of wash solution I (20 mM Tris-HCl, 500 mM NaCl, 20 mM imidazole, 3% glycerol, pH 8.0), 100 mL of wash solution II (20 mM Tris-HCl, 500 mM NaC1, 40 mM imidazole, 3% glycerol, pH 8.0), and 100 mL of wash solution III (20 mM Tris-HC1, 1 M NaCl, pH 8.0). A pre-cooled eluate (20 mM Tris-HCl, 150 mM NaC1, 500 mM imidazole, 10% glycerol, pH 8.0) was added to the well, which was placed vertically at 4°C for 10 min. The effluent was collected, and the wash was repeated once. The collected samples were subjected to SDS-PAGE, and the collected solution was concentrated and desalted according to the results.

**6. Protease activity validation of mutant proteins**

Synthesized HJ and 5' flap substrates containing 5' 6-FAM fluorescent dye (**Table 1**) were prepared and resuspended in annealing buffer (20 mM Tris-HCl pH 8.0, 50 mM NaCl, 0.1 mM EDTA). This solution was heated to 85°C, annealed for 5 min, and cooled slowly to room temperature. The GEN1 concentrations in the Holliday Junction reactions were 0, 8, 16, 32, 64, 128, and 256 nM. The GEN1 protein dosage gradient in the 5' flap reaction solutions were 0, 0.5, 1, 2, 4, 8, and 16 nM. Each reaction contained GEN1 WT or mutant proteins and 40 nM 6-FAM-labeled DNA substrate (HJ or 5' flap in 20 mM Tris-HCl pH 8.0, 50 ng/mL BSA, 1 mM DTT, and 5 mM MgCl_2_). After 15 min at 37°C, a buffer containing 15 mM EDTA and 0.3% SDS was added to stop the reaction. Using 1 mg/mL proteinase K, the reaction was deproteinized for 15 min at 37°C. Then, 10% Native-PAGE glue was used to separate and identify the cut products, and Typhoon FLA 7000 was used to capture and analyze the resulting images. The HJ and 5’ flap sequences are listed in **Table S2.**

**7. Electrophoretic mobility shift assay**

The following components were used in the reaction solutions (total 30 μL): l μL 40 ng/μL HJ DNA, 6 μL 5 × binding buffer (750 mM KC1, 0.5 mM DTT, 0.5 mM EDTA, 50 mM Tris, pH 7.4), and GEN1 WT or mutant protein proteins (final concentrations of 0, 37.5, 75, 150, or 300 nM), supplemented to 30 μL with ddH_2_O. Included in this assay were the following groups: 150 nM GEN1+cold competitor (×50), 150 nM GEN1+dsDNA (×1), and 300 nM BSA and 150 nM RAD52. For the negative control, 300 nM BSA was used, and for the positive control, 150 nM RAD52 was used. Each group was incubated at 37°C for 30 min. Then, 6× loading buffer was added to each solution. Electrophoresis was conducted using an 8% non-denaturing polyacrylamide gel and 0.5× TBE (44.5 mM Tris-HCl, 44.5 mM boric acid, 0.5 mM EDTA, pH 8.0) at 200 V for 4 h.

**8. Verification of mRNA splicing abnormalities**

WT and MT plasmids were transfected into HEK293 cells using Lipofectamine 2000 (Thermo Fisher Scientific). After culturing for 48 h, SNET+ proteinase K was added, and cultures were incubated at 55°C overnight to lyse the cells and extract cell DNA. KOD Dyemix (TOROIVD, KAO-201) was used to amplify the DNA using the following primers: pSPL3b-SD6-F2 CCTGGACACAACCTCAAAGGCA and pSPL3b-SA2-R2 ACCTGAGGAGTGAATTGGTCG. The amplification products were sent to Biotech for Sanger sequencing, using the same sequencing primers described above. Amplification primer electrophoresis was conducted at the same time and recorded using a developer (Tannon-2500r).
