# Supplementary material for: GEN1 as a risk factor for human congenital anomalies of the kidney and urinary tract
Source: Hum Genomics. 2024 Apr 24;18:41. doi: 10.1186/s40246-024-00606-8 (PMC11041010; doi:10.1186/s40246-024-00606-8)
Supplement: Supplementary file 2 — Supplementary Material 2 [file 40246_2024_606_MOESM2_ESM.docx]

**1.Mouse Gen1 (p.R400X) Point Mutation Project (CRISPR, oligo)**

## 1 Project summary

1. The mouse Gen1 gene (GenBank accession number: NM_177331.5; Ensembl: ENSMUSG00000051235) is located on mouse chromosome 12.
2. 14 exons have been identified, with the ATG start codon in exon 2 and the TAA stop codon in exon 14. The p.R400 is located on exon 11 and exon 12.
3. Exon 11 will be selected as target site (sequences shown on the next page).
4. gRNA targeting vector and donor oligo (with targeting sequence, flanked by 120 bp homologous sequences combined on both sides) will be designed.
5. The p. R400X (AGA to TGA) in donor oligo will be introduced into exon 11 by homology-directed repair. 2 synonymous mutations p.Q397= (CAG to CAA) and p.P398= (CCA to CCC) will also be introduced to prevent the binding and re-cutting of the sequence by gRNA after homology-directed repair.
6. Cas9, gRNA and donor oligo will be co-injected into fertilized eggs for KI mouse production.
7. The pups will be genotyped by PCR followed by sequence analysis.

### 1.2 Schematic depiction of targeting strategy


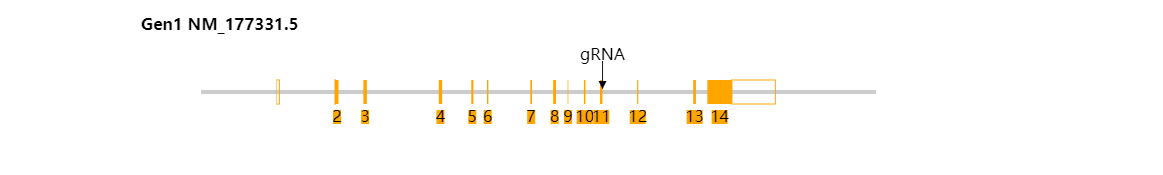
Genomic region of mouse Gen1 locus is diagrammed below. Solid bars represent ORF; open bars present UTRs.

Wildtype allele before targeting:

#### GCTTTTGACCCGCTATGACATGATAGAAAGAAAACATGGTAGAAAGACCTCCAATC//AACTA(CAGCCA)ATTAG GTAAT//GTATGGTTGACACTGGAGACATGGCTCAGCAGTTAAGAATAACTGTTGCTCTG

*Note: “//” indicates gRNA cut site.*

Mutant allele after targeting:

#### GCTTTTGACCCGCTATGACATGATAGAAAGAAAACATGGTAGAAAGACCTCCAATCAACTA(CAACCC)ATTTGGT AATGTATGGTTGACACTGGAGACATGGCTCAGCAGTTAAGAATAACTGTTGCTCTG

*Note: Sequence in parentheses in wildtype allele will be replaced by sequence in parentheses in mutation allele after successful targeting.*

### 1.3 gRNA target sequence

gRNA-A1 (matches forward strand of gene): ACAGCCAATTAGGTAATGTA-TGG

gRNA-B1 (matches reverse strand of gene): CCTAATTGGCTGTAGTTGAT-TGG

*Note: Color schemes are employed to facilitate tracking of sequences.*

### 1.4 Donor oligo sequence

Donor oligo

#### GCTTTTGACCCGCTATGACATGATAGAAAGAAAACATGGTAGAAAGACCTCCAATCAACTACAACCCATTTGGTAA TGTATGGTTGACACTGGAGACATGGCTCAGCAGTTAAGAATAACTGTTGCTCTG

*Note: The mutation sequences is colored in red, synonymous mutation sequences is colored in Magenta.*

### 1.5 Assay of CRISPR-induced mutation

The target region of mouse Gen1 locus will be amplified by PCR with specific primers. PCR product will be sequenced to confirm targeting.

Primer sequence:

Primer-F: TACATAGACTGGTACTCTGGTGG

Primer-R: GGGAGCAACACTTCTGATTATC

**1.6 PCR Screening**

**PCR Primers (Annealing Temperature 60.0 ºC):**

Forward primer (F1): 5’-TGTCATTTGACACCTTCTCCTTAG-3’

Reverse primer (R1): 5’-CCATAGGGAGCAACACTTCTGAT-3’

**PCR Results:**

F1 animals 1, 3 and 5 and other animals amplified by PCR, the PCR product will be used to sequencing

confirmation.

**Marker Positive F1 (MT: 673 bp; WT: 673 bp)**


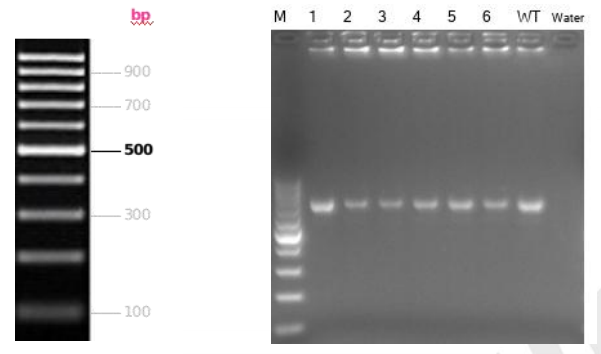


**1.7 Sequencing Confirmation**

**Sequencing Primer:**

F1: 5’-TGTCATTTGACACCTTCTCCTTAG-3’

**Sequencing Results:**

F1 animals 1, 3 and 5 With A to T mutation and silent mutations (p.Q397= (CAG to CAA) and p.P398= (CCA

to CCC)), Exons underlined.

**Mouse ID:1, 3, 5**

Wildtype：GAAAGAAAACATGGTAGAAAGACCTCCAATCAACTACAGCCAATTAGGTAATGTATGGTTGACACTGGAGACATGGCTCA

Mutation：GAAAGAAAACATGGTAGAAAGACCTCCAATCAACTACAACCCATTTGGTAATGTATGGTTGACACTGGAGACATGGCTCA


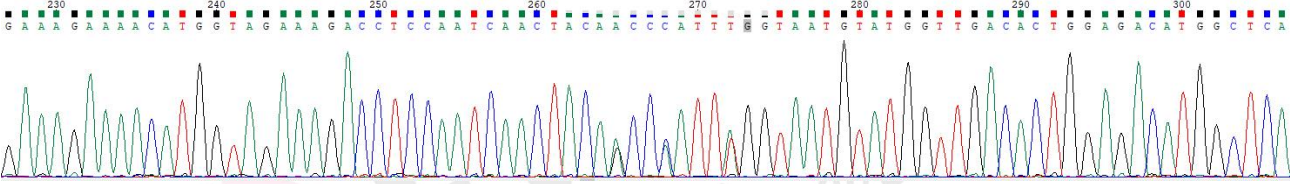


**1.8 Breeding and Genotyping strategy**

Inter-cross heterozygous targeted mice to generate homozygous targeted mice

Primers:

F1: 5’-TGTCATTTGACACCTTCTCCTTAG-3’

R1: 5’-CCATAGGGAGCAACACTTCTGAT-3’

PCR product size 673 bp

The 673 bp PCR product will be used to sequencing confirmation.

Sequencing Primer:

F1: 5’-TGTCATTTGACACCTTCTCCTTAG-3’

Sequencing Confirmation:

Homozygotes:

MT:

GAAAGAAAACATGGTAGAAAGACCTCCAATCAACTACAACCCATTTGGTAATGTATGGTTGACACTGGAGACATGGCTCA

Heterozygotes:

MT:

GAAAGAAAACATGGTAGAAAGACCTCCAATCAACTACAACCCATTTGGTAATGTATGGTTGACACTGGAGACATGGCTCA

WT:

GAAAGAAAACATGGTAGAAAGACCTCCAATCAACTACAGCCAATTAGGTAATGTATGGTTGACACTGGAGACATGGCTCA

Wildtype allele:

WT:

GAAAGAAAACATGGTAGAAAGACCTCCAATCAACTACAGCCAATTAGGTAATGTATGGTTGACACTGGAGACATGGCTCA

**2.Mouse Gen1 (p.T105R) Point Mutation Project (CRISPR, oligo)**

## 2.1 Project summary

1. The mouse Gen1 gene (GenBank accession number: NM_177331.5; Ensembl: ENSMUSG00000051235) is located on mouse chromosome 12.
2. 14 exons have been identified, with the ATG start codon in exon 2 and the TAA stop codon in exon 14. The p.T105 is located on exon 3.
3. Exon 3 will be selected as target site (sequences shown on the next page).
4. gRNA targeting vector and donor oligo (with targeting sequence, flanked by 120 bp homologous sequences combined on both sides) will be designed.
5. The p.T105R (ACA to AGA) in donor oligo will be introduced into exon 3 by homology-directed repair. 1 synonymous mutation p.R101= (AGG to CGT) will also be introduced to prevent the binding and re-cutting of the sequence by gRNA after homology-directed repair.
6. Cas9, gRNA and donor oligo will be co-injected into fertilized eggs for KI mouse production.
7. The pups will be genotyped by PCR followed by sequence analysis.

### 2.2 Schematic depiction of targeting strategy


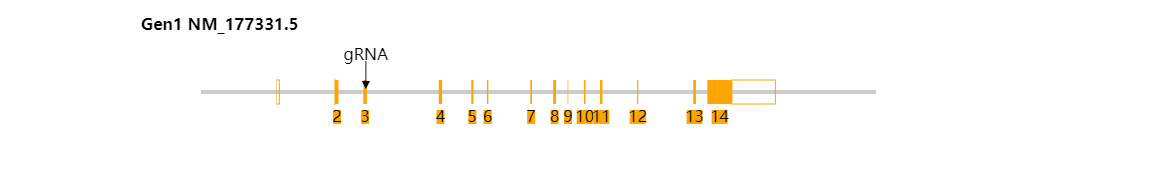
Genomic region of mouse Gen1 locus is diagrammed below. Solid bars represent ORF; open bars present UTRs.

Wildtype allele before targeting:

#### CTGAAAGCTGATGTCATAAGCAAGAGGACTCAGACTCGTTACGGGCCTTCTGGAAAA//TCA(AGG)TCTCAGAA/

/A(ACA)GGGAGATCACATTTTAAGTCAGTCTTACGAGAGGTGAGCATTCAGATTCGACTAGTAATT

*Note: “//” indicates gRNA cut site.*

Mutant allele after targeting:

#### CTGAAAGCTGATGTCATAAGCAAGAGGACTCAGACTCGTTACGGGCCTTCTGGAAAATCA(CGT)TCTCAGAAA(A GA)GGGAGATCACATTTTAAGTCAGTCTTACGAGAGGTGAGCATTCAGATTCGACTAGTAATT

*Note: Sequence in parentheses in wildtype allele will be replaced by sequence in parentheses in mutation allele after successful targeting.*

### 2.3 gRNA target sequence

gRNA-A1 (matches forward strand of gene): ACGGGCCTTCTGGAAAATCA-AGG

gRNA-B1 (matches forward strand of gene): AAATCAAGGTCTCAGAAAAC-AGG

*Note: Color schemes are employed to facilitate tracking of sequences.*

### 2.4 Donor oligo sequence

Donor oligo

#### CTGAAAGCTGATGTCATAAGCAAGAGGACTCAGACTCGTTACGGGCCTTCTGGAAAATCACGTTCTCAGAAAAGAG GGAGATCACATTTTAAGTCAGTCTTACGAGAGGTGAGCATTCAGATTCGACTAGTAATT

*Note: The mutation sequences is colored in red, synonymous mutation sequences is colored in Magenta.*

### 2.5 Assay of CRISPR-induced mutation

The target region of mouse Gen1 locus will be amplified by PCR with specific primers. PCR product will be sequenced to confirm targeting.

Primer sequence:

Primer-F: TGCTAGTATATGCAATGGTGTC

Primer-R: TCACTGCGGAGTATTACTATGTC

**2.6 PCR Screening**

**PCR Primers (Annealing Temperature 60.0 ºC):**

Forward primer (F1): 5’-CCTAGTATTTTGTACTCCTATTGGGTC-3’

Reverse primer (R1): 5’-CACTGCGGAGTATTACTATGTCCA-3’

**PCR Results:**

F1 animals 4, 5, 6 and 7 and other animals amplified by PCR, the PCR product will be used to sequencing

confirmation.

**Marker Positive F1 (MT: 492 bp; WT: 492 bp)**


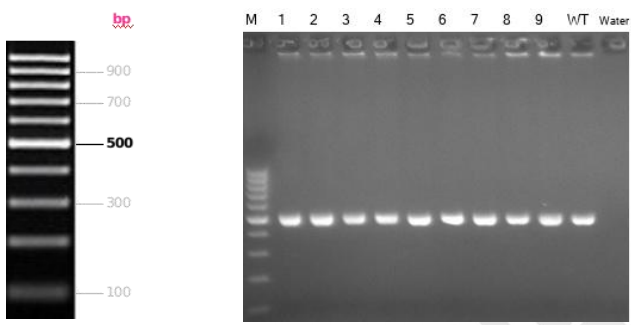


**Sequencing Primer:**

R1: 5’-CACTGCGGAGTATTACTATGTCCA-3’

**Sequencing Results:**

F1 animals 4, 5, 6 and 7 With p.T105R (ACA to AGA) mutation and silent mutation p.R101= (AGG to CGT)

**Mouse ID:4, 5, 6, 7**

Wildtype：ACTCGTTACGGGCCTTCTGGAAAATCAAGGTCTCAGAAAACAGGGAGATCACATTTTAAGTCAGTCTTACGAGAGGTGAG

Mutation：ACTCGTTACGGGCCTTCTGGAAAATCACGTTCTCAGAAAAGAGGGAGATCACATTTTAAGTCAGTCTTACGAGAGGTGAG


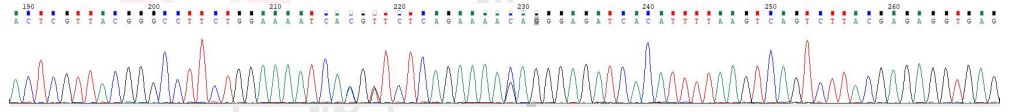


**2.7 Breeding and Genotyping strategy**

Inter-cross heterozygous targeted mice to generate homozygous targeted mice

Primers:

F1: 5’-CCTAGTATTTTGTACTCCTATTGGGTC-3’

R1: 5’-CACTGCGGAGTATTACTATGTCCA-3’

PCR product size 492 bp

The 492 bp PCR product will be used to sequencing confirmation.

Sequencing Primer:

R1: 5’-CACTGCGGAGTATTACTATGTCCA-3’

Sequencing Confirmation:

Homozygotes:

MT:ACTCGTTACGGGCCTTCTGGAAAATCACGTTCTCAGAAAAGAGGGAGATCACATTTTAAGTCAGTCTTACGAGAGGTGAG

Heterozygotes:

MT: ACTCGTTACGGGCCTTCTGGAAAATCACGTTCTCAGAAAAGAGGGAGATCACATTTTAAGTCAGTCTTACGAGAGGTGAG

WT: ACTCGTTACGGGCCTTCTGGAAAATCAAGGTCTCAGAAAACAGGGAGATCACATTTTAAGTCAGTCTTACGAGAGGTGAG

Wildtype allele:

WT: ACTCGTTACGGGCCTTCTGGAAAATCAAGGTCTCAGAAAACAGGGAGATCACATTTTAAGTCAGTCTTACGAGAGGTGAG

**3.Mouse Gen1 (c.1068+3A to G) Point Mutation Project (CRISPR, oligo)**

## 3.1 Project summary

1. The mouse Gen1 gene (GenBank accession number: NM_177331.5; Ensembl: ENSMUSG00000051235) is located on mouse chromosome 12.
2. 14 exons have been identified, with the ATG start codon in exon 2 and the TAA stop codon in exon 14. The c.1068+3A is located on intron10.
3. Intron10 will be selected as target site (sequences shown on the next page).
4. gRNA targeting vector and donor oligo (with targeting sequence, flanked by 120 bp homologous sequences combined on both sides) will be designed.
5. The c.1068+3A to G in donor oligo will be introduced into intron 10 by homology-directed repair. 1 synonymous mutation p.L354= (TTG to CTC) will also be introduced to prevent the binding and re-cutting of the sequence by gRNA after homology-directed repair.
6. Cas9, gRNA and donor oligo will be co-injected into fertilized eggs for KI mouse production.
7. The pups will be genotyped by PCR followed by sequence analysis.

### 3.2 Schematic depiction of targeting strategy

Genomic region of mouse Gen1 locus is diagrammed below. Solid bars represent ORF; open bars present UTRs.


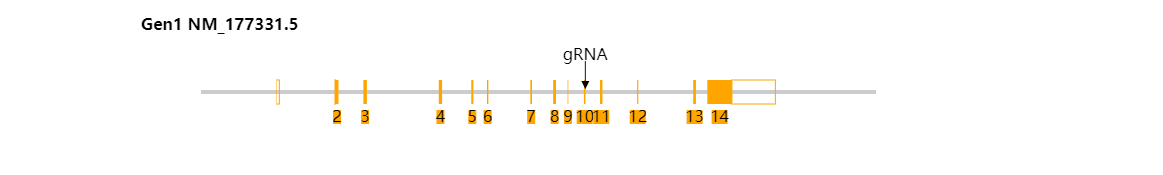


Wildtype allele before targeting:

#### TTCCTTTTGAATAAGAATAAAATGTTGAAACCGATCACATACCAAAGACCTGATTTATTA(TTG)T//TTCAGGT/

/ATCTGGAAGTAAATTCTTACTTAATAATACAAAATTAGATATGAGAAAGATCAAAAGG

*Note: “//” indicates gRNA cut site.*

Mutant allele after targeting:

#### TTCCTTTTGAATAAGAATAAAATGTTGAAACCGATCACATACCAAAGACCTGATTTATTA(CTC)TTTCAGGTGTC TGGAAGTAAATTCTTACTTAATAATACAAAATTAGATATGAGAAAGATCAAAAGG

*Note: Sequence in parentheses in wildtype allele will be replaced by sequence in parentheses in mutation allele after successful targeting.*

### 3.3 gRNA target sequence

gRNA-A1 (matches forward strand of gene): ACCTGATTTATTATTGTTTC-AGG

gRNA-B1 (matches forward strand of gene): TTATTATTGTTTCAGGTATC-TGG

*Note: Color schemes are employed to facilitate tracking of sequences.*

### 3.4 Donor oligo sequence

Donor oligo

#### TTCCTTTTGAATAAGAATAAAATGTTGAAACCGATCACATACCAAAGACCTGATTTATTACTCTTTCAGGTGTCTG GAAGTAAATTCTTACTTAATAATACAAAATTAGATATGAGAAAGATCAAAAGG

*Note: The mutation sequences is colored in red, synonymous mutation sequences is colored in Magenta.*

### 3.5 Assay of CRISPR-induced mutation

The target region of mouse Gen1 locus will be amplified by PCR with specific primers. PCR product will be sequenced to confirm targeting.

Primer sequence:

Primer-F: AGCCAGATTAGATCAGTCAGTT

Primer-R: CATAGTAACCGAGACATCTTTC

**3.6** **PCR Screening**

**PCR Primers (Annealing Temperature 60.0 ºC):**

Forward primer (F1): 5’-GTAGGCAGTAGAATTGAGTTCATGG-3’

Reverse primer (R1): 5’-GTCTGCACATAGTAACCGAGACAT-3’

**PCR Results:**

F1 animals 7, 8 and 12 and other animals amplified by PCR, the PCR product will be used to sequencing

confirmation.

**Marker Positive F1 (MT: 831 bp; WT: 831 bp)**


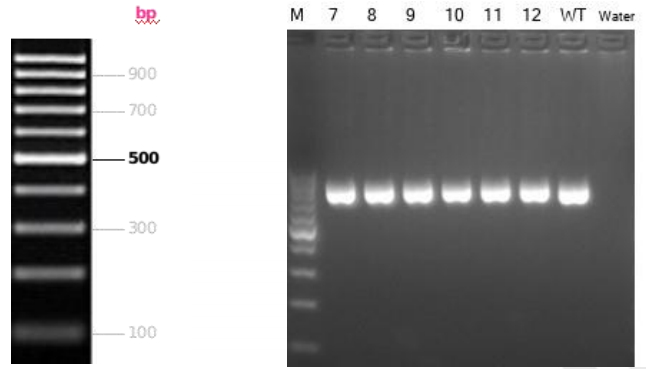


**Sequencing Primer:**

R1: 5’-GTCTGCACATAGTAACCGAGACAT-3’

**Sequencing Results:**

F1 animals 7, 8 and 12 With mutation (c.1068+3A to G) and silent mutation (TTG to CTC)

**Mouse ID:7, 8, 12**

Wildtype：ATGTTGAAACCGATCACATACCAAAGACCTGATTTATTATTGTTTCAGGTATCTGGAAGTAAATTCTTACTTAATAATAC

Mutation：ATGTTGAAACCGATCACATACCAAAGACCTGATTTATTACTCTTTCAGGTGTCTGGAAGTAAATTCTTACTTAATAATAC


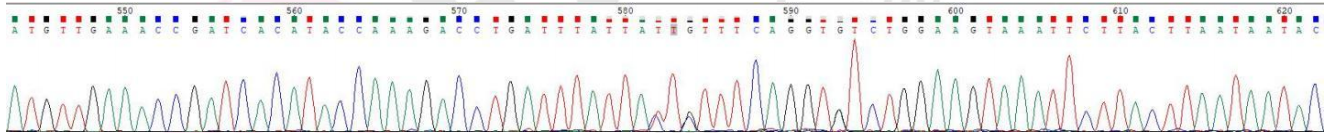


**3.7 Breeding and Genotyping strategy**

Inter-cross heterozygous targeted mice to generate homozygous targeted mice

Primers:

F1: 5’-GTAGGCAGTAGAATTGAGTTCATGG-3’

R1: 5’-GTCTGCACATAGTAACCGAGACAT-3’

PCR product size 831 bp

The 831 bp PCR product will be used to sequencing confirmation.

Sequencing Primer:

R1: 5’-GTCTGCACATAGTAACCGAGACAT-3’

Sequencing Confirmation:

Homozygotes:

MT:

ATGTTGAAACCGATCACATACCAAAGACCTGATTTATTACTCTTTCAGGTGTCTGGAAGTAAATTCTTACTTAATAATAC

Heterozygotes:

MT:

ATGTTGAAACCGATCACATACCAAAGACCTGATTTATTACTCTTTCAGGTGTCTGGAAGTAAATTCTTACTTAATAATAC

WT:

ATGTTGAAACCGATCACATACCAAAGACCTGATTTATTATTGTTTCAGGTATCTGGAAGTAAATTCTTACTTAATAATAC

Wildtype allele:

WT:

ATGTTGAAACCGATCACATACCAAAGACCTGATTTATTATTGTTTCAGGTATCTGGAAGTAAATTCTTACTTAATAATAC
