# Supplementary material for: GEN1 as a risk factor for human congenital anomalies of the kidney and urinary tract
Source: Hum Genomics. 2024 Apr 24;18:41. doi: 10.1186/s40246-024-00606-8 (PMC11041010; doi:10.1186/s40246-024-00606-8)
Supplement: Supplementary file 5 — Supplementary Material 5 [file 40246_2024_606_MOESM5_ESM.docx]

# SUPPLEMENTARY TABLES

**Table S1.** Mutation site information, mutant primer sequences and sequencing primers.

**Table S2.** Sequence of HJ and 5FLAP.

**Table S3.** Point mutant mouse gene detection sequence.

**Table S1.** Mutation site information, mutant primer sequences and sequencing primers.

| **Variant sites (GEN1)** | **mutant primer sequences 5`-3`** |
| --- | --- |
| **c.445G>A** | **F: aatggctgcctcaccaatgatggagata** |
|  | **R: gacatgaccaccagcattgagataagca** |
| **c.1201C>T** | **F: tgaattgttaagactcgaatcagaa** |
|  | **R: aattggctgtagttgattagagttt** |
| **c.1657A>G** | **F: gtacagcaaattaaagctgtcagtaagt** |
|  | **R: agccaaaggtcttagagaagacatga** |
| **c.2116T>C** | **F: ccttgtattgctaacagtggttctg** |
|  | **R: ttcttttacactaagtatggacaaag** |
| **c.2527C>T** | **F: tctaagatacatattaaagaaactgaac** |
|  | **R: gctactcaacttgttatggccactttc** |
| **c.314C>G** | **F: gagggagatcacattttaaatcagtc** |
|  | **R: ttttctgagaccacgattttccaga** |
| **c.1730T>C** | **F: catccgtgattgctgatctacacttgag** |
|  | **R: tattatgagatgaggtattgggttgactag** |
| **c.1609T>C** | **F: cgtttgaatgcacaagaacagttcatgtc** |
|  | **R: tggagtatttttaggtaaaagcaagct** |
| **c.1106A>T** | **F: tctatgcatgtgagaaattgctgg** |
|  | **R: gattgggccactccatttttt** |
| **Sequencing primes** | **5`-3`** |
| **F1** | **ACACATCCCCTTGCGTAATC** |
| **R1** | **CCGAGACTGATTCCTCTTGC** |
| **F2** | **TCCCTGGAGTTGGAAAAGAG** |
| **F3** | **GGAGTGGCCCAATCACTATG** |
| **F4** | **GAGCACTATTGACTGGGAAGG** |
| **F5** | **TTCCCTTGCAAAATGAATCC** |
| **F6** | **TGGCCATAACAAGTTGAGTAGC** |

**Table S2.** Sequence of HJ and 5’FLAP.

CB209、CB210、CB211、CB212 formed Holliday Junction；CB209、CB212、CB218 formed 5’ flaps.HJ-1,HJ-2,HJ-3,HJ-4 forms a 12 bp homology region. The 5' end of HJ-1 is pre-tagged with T4 polynucleotide kinase and γ- 32P-ATP, and then annealed along with the remaining 3 unlabeled chains.

| **Oligonucleotide** | **Sequence (5’-3’)** |
| --- | --- |
| **CB209 (5’ 6FAM)** | **ACGCTGCCGAATTCTACCAGTGCCTTGCTAGGACATCTTTGCCCACCTGCAGGTTCACCC** |
| **CB210** | **GGGTGAACCTGCAGGTGGGCAAAGATGTCCATCTGTTGTAATCGTCAAGCTTTATGCCGT** |
| **CB211** | **ACGGCATAAAGCTTGACGATTACAACAGATCATGGAGCTGTCTAGAGGATCCGACTATCG** |
| **CB212** | **CGATAGTCGGATCCTCTAGACAGCTCCATGTAGCAAGGCACTGGTAGAATTCGGCAGCGT** |
| **CB218** | **GGGTGAACCTGCAGGTGGGCAAAGATGTCC** |
| **HJ-1** | **ATCGATGTCTCTAGACAGCTGCTCAGGATTGATCTGTAATGGCCTGGGA** |
| **HJ-2** | **GTCCCAGGCCATTACAGATCAATCCTGAGCATGTTTACCAAGCGCATTG** |
| **HJ-3** | **TGATCACTTGCTAGCGTCGCAATCCTGAGCAGCTGTCTAGAGACATCGA** |
| **HJ-4** | **CCAATGCGCTTGGTAAACATGCTCAGGATTGCGACGCTAGCAAGTGATC** |

**Table S3.** Point mutant mouse gene detection sequence.

| **KICMPS220211XB1-Gen1 (c.1068+3A to G)-NM2822:（5’-3’）** | |
| --- | --- |
| **E1196-NM2822- F1:** | **GTAGGCAGTAGAATTGAGTTCATGG** |
| **E1201-NM2822- R1:** | **GTCTGCACATAGTAACCGAGAACAT** |
| **KICMPS220211XB2-Gen1 (p.R400X)- NM2823:** | |
| **E1192-NM2823-F1:** | **TGTCATTTGACACCTTCTCCTTAG** |
| **E1193-NM2823-R1:** | **CCATAGGGAGCAACACTTCTGAT** |
| **KICMPS220211XB3-Gen1 (p.T105R)-NM2824:** | |
| **E1189-NM2824-F1:** | **CCTAGTATTTTGTACTCTATTGGGTC** |
| **E1190-NM2824-R1:** | **CACTGCGGAGTATTACTATGTCCA** |
