# Supplementary material for: GEN1 as a risk factor for human congenital anomalies of the kidney and urinary tract
Source: Hum Genomics. 2024 Apr 24;18:41. doi: 10.1186/s40246-024-00606-8 (PMC11041010; doi:10.1186/s40246-024-00606-8)
Supplement: Supplementary file 6 — Supplementary Material 6 [file 40246_2024_606_MOESM6_ESM.docx]

**Figure S1.** The plasmid structure diagram.

**Figure S2.** The research route of this study.

**Figure S3.** Mutation sites distribution on the GEN1 protein domain (NP_872431).

**Figure S4.** DNA binding ability of WT and mutated GEN1 proteins.


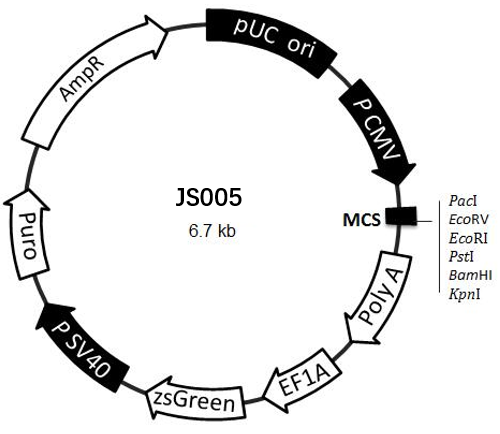

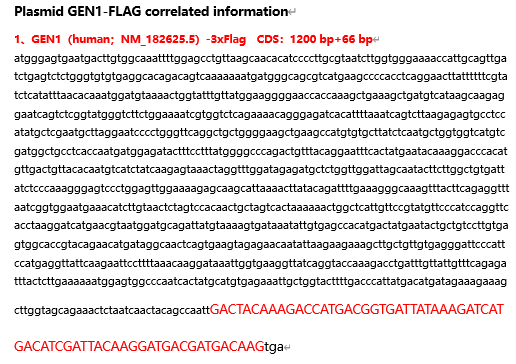

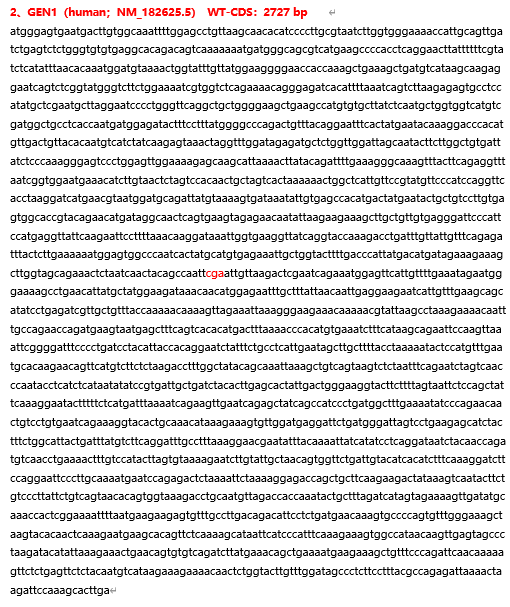


**Figure S1. The sequence and plasmid structure diagram.**

The GEN1 point mutated plasmids used in this study were all based on JS005, inserted into the GEN1 cDNA (NM_182625.5), and FLAG was used as the protein expression tail, so that the expressed GEN1 protein was tagged with FLAG, which was conducive to the later experiments and the evaluation of results**.** At the same time, JS005 also expresses green fluorescent protein, which makes the plasmid transcriptional expression efficiency clearly observed in cells. For truncated mutation GEN1 (p.R401X,508), the GEN1-FLAG base sequence in the plasmid is as GEN1 (human; NM_182625.5)-3xFLAG CDS:1200bp+60bp showed.


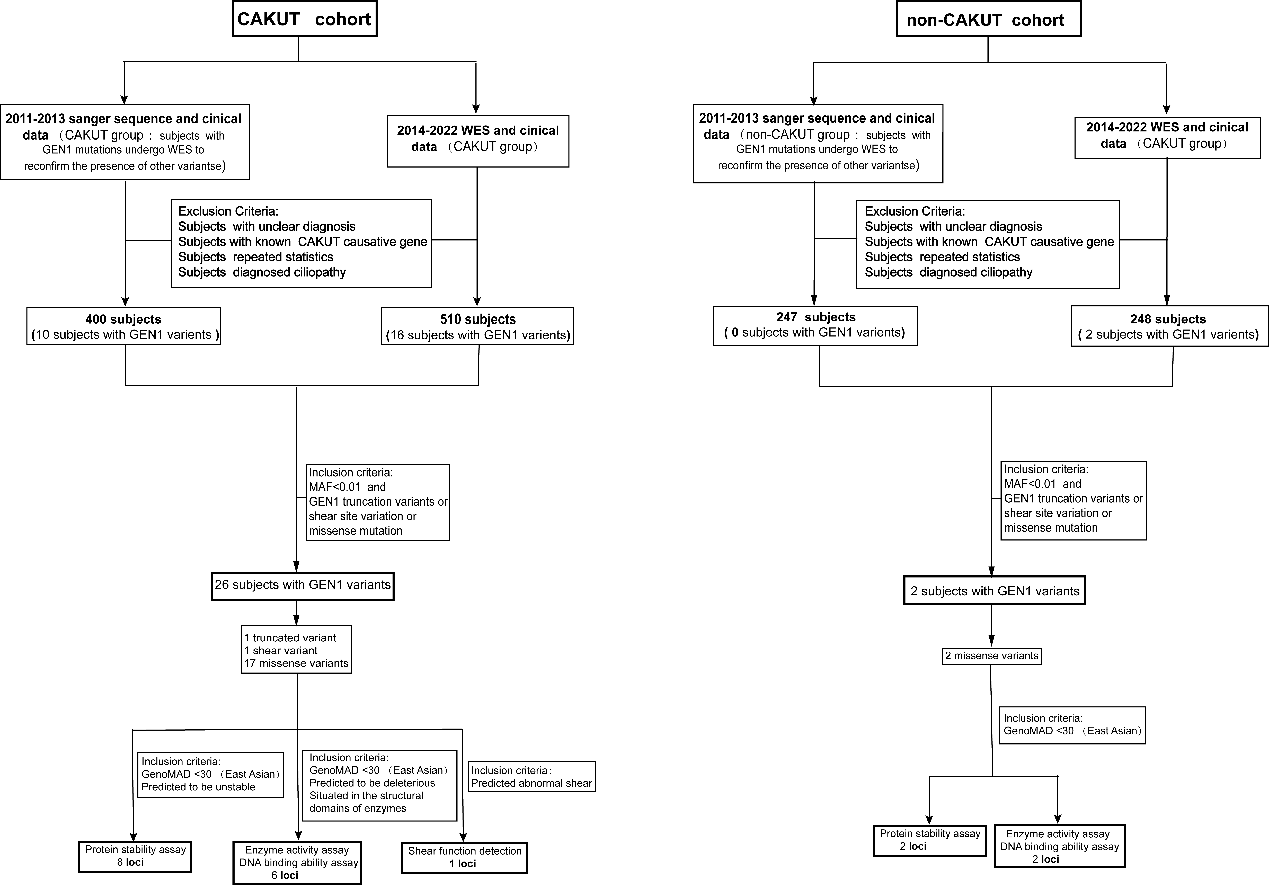


**Figure S2. The research route of this study.**

The above process demonstrates the inclusion and exclusion criteria for the CKUT and non-CAKUT groups in this study. The inclusion of the two groups of population was divided into two parts according to the year, in which the gene variation data from 2011 to 2013 were from Sanger sequence, and the gene variation data from 2014 to 2022 were from WES. For samples with GEN1 variants detected with Sanger in 2011-2013, we further confirmed with WES that there were no other known CAKUT pathogenic gene variants. Exclusion criterions include subjects with unclear diagnosis, other known CAKUT causative gene variants, duplicate statistics, and subjects diagnosed with ciliopathies. For the definition of the GEN1 variant, we identified the truncation, missense or shear site variant of MAF<0.01 as a GEN1 variant. Protein stability assay was performed for the screened GEN1 variants, which GenoMAD<30 (East Asian) and predicted to be unstable. Variants which GenoMAD<30 (East Asian) and predicted to be a deleterious variant or located in the structural domains of enzymes was tested for enzyme activity assay. The splice site was performed shear function detection. For the two sites in the control group, protein stability experiments and enzyme activity assay were carried out as controls.


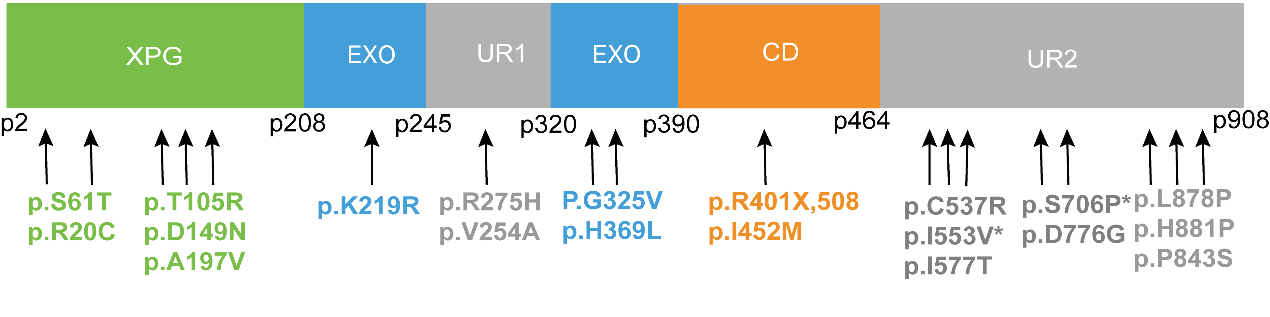


**Figure S3. Mutation sites distribution on the GEN1 protein domain (NP_872431).**

The above image represents a simplified diagram of GEN1 protein, which is composed of 908 amino acids and is divided into 4 regions and 6 parts according to function. The colored regions correlate with enzyme activity, and the gray regions’ function is not clear. The distributions of the variant sites involved in this study are annotated at the bottom of the figure. Abbreviations: XPG-N, N-terminal domain; XPG-I, internal domain; EXO, Exonuclease domain; UR, Unknown region; CD, Chromatin domain. * sites in the non-CAKUT group.


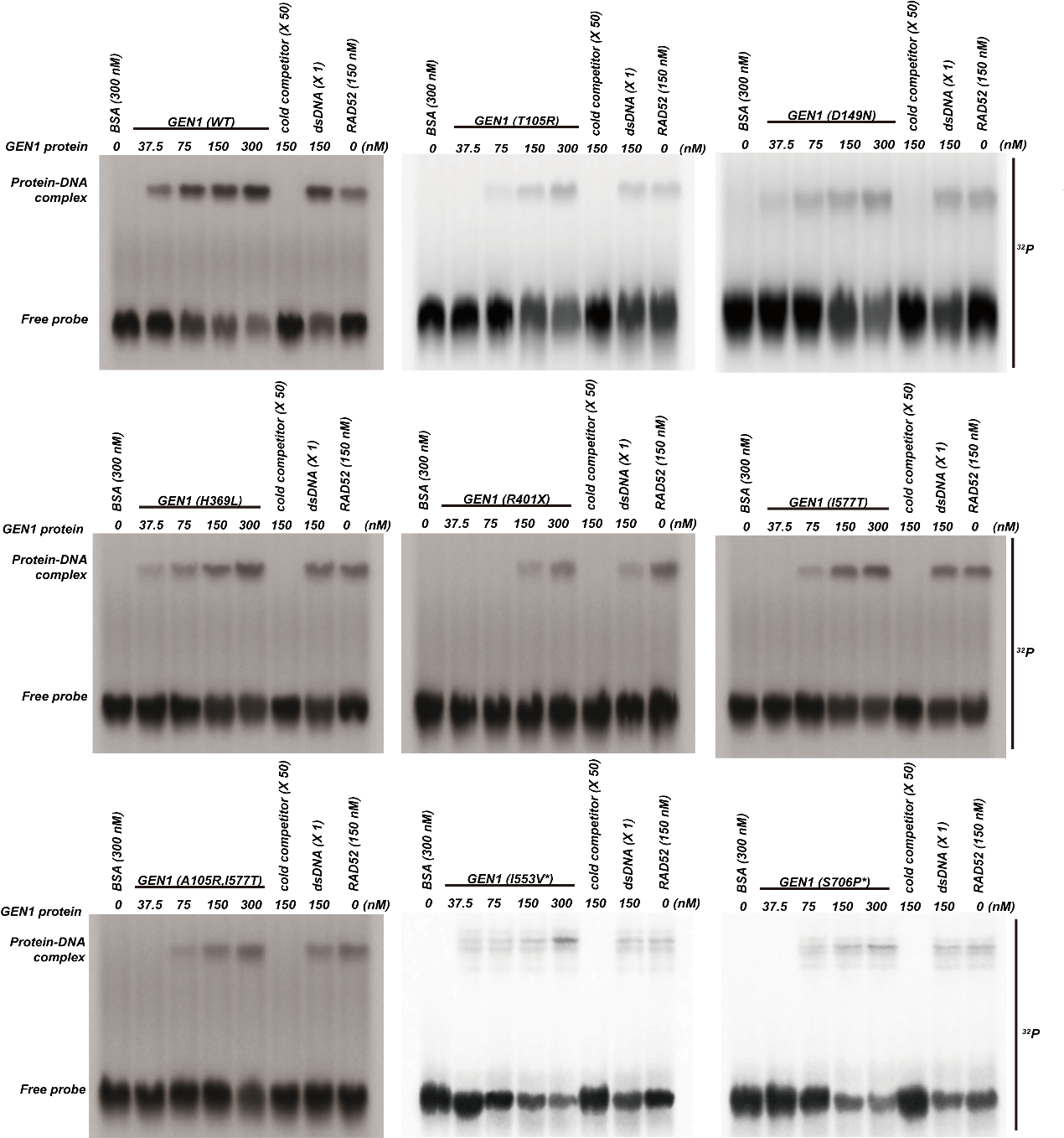


**Figure S4. DNA binding ability of WT and mutated GEN1 proteins.**

EMSA was conducted to determine the binding ability of WT and mutant GEN1 proteins to DNA (HJ). DNA protein binding capacity was assessed based on the minimum protein concentration that binds to DNA. Five different protein concentrations were applied in this experiment to distinguish DNA binding capacity. All the results of all mutated proteins are evaluated from the baseline results of wild-type proteins.

* Sites in non-CAKUT group. Abbreviations: EMSA, electrophoretic mobility shift assay; HJ, Holliday junction; WT, wild type.
